# Sustained cancer‐relevant alternative RNA splicing events driven by PRMT5 in high‐risk neuroblastoma

**DOI:** 10.1002/1878-0261.13702

**Published:** 2024-07-17

**Authors:** Laurel Tabe Bate‐Eya, Gulsah Albayrak, Simon Mark Carr, Amit Shrestha, Alexander Kanapin, Anastasia Samsonova, Nicholas Barrie La Thangue

**Affiliations:** ^1^ Laboratory of Cancer Biology, Department of Oncology University of Oxford UK; ^2^ Institute of Translational Biomedicine Saint Petersburg State University Russia

**Keywords:** alternative RNA splicing, apoptosis, E2F1, neuroblastoma, PRMT5, therapeutic target

## Abstract

Protein arginine methyltransferase 5 (*PRMT5*) is over‐expressed in a wide variety of cancers and is implicated as having a key oncogenic role, achieved in part through its control of the master transcription regulator E2F1. We investigated the relevance of PRMT5 and E2F1 in neuroblastoma (NB) and found that elevated expression of *PRMT5* and *E2F1* occurs in poor prognosis high‐risk disease and correlates with an amplified Myelocytomatosis viral‐related oncogene, neuroblastoma‐derived (*MYCN*) gene. Our results show that MYCN drives the expression of splicing factor genes that, together with PRMT5 and E2F1, lead to a deregulated alternative RNA splicing programme that impedes apoptosis. Pharmacological inhibition of PRMT5 or inactivation of E2F1 restores normal splicing and renders NB cells sensitive to apoptosis. Our findings suggest that a sustained cancer‐relevant alternative RNA splicing programme desensitises NB cells to apoptosis, and identify PRMT5 as a potential therapeutic target for high‐risk disease.

## Graphical abstract

High expression of *MYCN*, *PRMT5* and *E2F1* in neuroblastoma cells derived from high‐risk disease causes a deregulated alternative RNA splicing programme and resistance to cell death. The pharmacological inhibition of PRMT5, or E2F1 inactivation, restores normal splicing, rendering neuroblastoma cells sensitive to apoptosis. Our findings therefore identify PRMT5 as a potential therapeutic target for the treatment of high‐risk neuroblastoma.

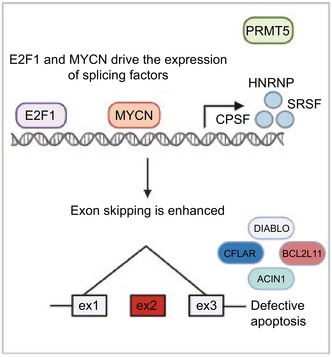

**Abbreviations**
ACIN1: apoptotic chromatin condenser inducer 1
CFLAR: CASP8 and FADD like apoptosis regulator
ChIP: chromatin immunoprecipitation
Cr: CRISPR
DEGs: differentially expressed genes
E2F1: E2 promoter binding factor 1
FACS: flourescence activated cell sorting
GEO: Gene Expression Omnibus
GFP: green fluorescence protein
GO: Gene Ontology
HA: Haemagglutinin
INSS: international neuroblastoma staging system
KEGG: Kyoto Encyclopedia of Genes and Genomes
MTT: 3‐[4,5‐dimethylthiazol‐2‐yl]‐2,5 diphenyl tetrazolium bromide
MYCN: myelocytomatosis viral‐related oncogene, neuroblastoma derived
NB: neuroblastoma
ORF: open reading frame
OS: overall survival
pRb: retinoblastoma protein
PRMT5: protein arginine methyltransferase 5
PSI: per cent spliced in
rMATS: replicate multivariate analysis of transcript splicing
TARGET: therapeutically applicable research to generate effective treatments
TCGA: The Cancer Genome Atlas
Vst: variance stabilising transformation
WT: wild type

## 1 Introduction

Neuroblastoma is the most common extracranial solid tumour in children and is associated with significant morbidity and mortality [1]. The disease exhibits phenotypic heterogeneity and is broadly divided into poor prognosis high‐risk and improved prognosis lower‐risk disease [2], and metastatic drug‐resistant disease is a major clinical challenge [3]. One of the major obstacles in developing new treatment modalities is the limited understanding of the biologically relevant cancer pathways that drive the malignant phenotype [2]. A variety of somatic mutations in oncogenic pathways have been described, for example, amplification of the *MYCN* gene and mutation in the *ALK* and *ATRX* genes [4, 5, 6, 7] *MYCN* gene amplification occurs in about 25% of tumours and is a common feature of high‐risk disease, with rapid disease progression and poor prognosis; abnormal *MYCN* expression is the strongest predictor of poor prognosis [8]. In contrast, germline mutation in the *ALK* gene is a predisposition marker for familial neuroblastoma, which also undergoes somatic mutations in sporadic cases [9]. A variety of other somatic mutations have been described in genes encoding chromatin‐associated proteins [5, 9, 10].

The E2F family of transcription factors play an important role in various cellular processes such as DNA replication and repair, apoptosis, cell differentiation, the cell cycle and most recently alternative splicing [11, 12, 13]. E2F1 is the most widely studied member of the E2F family which, through its control by the retinoblastoma protein (pRb), is important in the regulation of cell cycle progression in diverse cell types, with deregulation of the pathway being one of the hall marks for cancer [11]. However, the E2F pathway regulates a much larger gene network than originally envisaged, reflecting in part the role of arginine methylation, mediated by PRMTs including PRMT5 [14, 15, 16]. Residue‐specific methylation occurs within a central arginine (R)‐rich cluster in E2F1 which at the biological level facilitates proliferation and cell growth [15, 16] and coincides with a switch in E2F1 activity, from its primary role as a transcriptional regulator to one with a broad influence on gene expression, including non‐transcription processes like alternative RNA splicing [13]. The methylation event is read by the Tudor domain‐containing protein, p100/Tudor staphylococcal nuclease (p100/TSN), which enables E2F1 activity to be coordinated with the RNA splicing machinery [13, 16]. The frequent over‐expression of *PRMT5* in diverse human tumours, which includes neuroblastoma [17], and the critical role ascribed to E2F1 in the cancer cell cycle [11] suggests that PRMT5 and E2F1 may play an important role in driving the malignant phenotype.

Here, we wanted to elucidate the role that PRMT5, E2F1 and MYCN may take on in neuroblastoma. We found that high levels of *PRMT5* and *E2F1* expression were apparent in clinical biopsies taken from poor prognosis high‐risk disease which coincided with *MYCN* amplification. In NB cell lines that recapitulated a similar high expression profile, we found a sustained increase in alternative RNA splicing activity. Mechanistically, MYCN transcriptionally activated genes encoding splicing factors which together with PRMT5 and E2F1 fostered a cellular environment that led to exon‐skipping events in genes required for apoptosis. This led to the synthesis of protein isoforms that were compromised in apoptotic activity. Most importantly, pharmacological inhibition of PRMT5 reinstated a splicing programme that enhanced apoptosis in high expression profile NB cells. These results uncover an unexpected relationship between MYCN, PRMT5 and E2F1 that leads to a sustained and deregulated alternative RNA splicing programme which confers a reduced sensitivity to apoptosis. Our results further highlight PRMT5 as a possible therapeutic target in high‐risk NB.

## 2 Materials and methods

### 2.1 Cell culture, cell line generation and compound treatment

#### 2.1.1 Neuroblastoma cells

CHP‐134 (RRID:CVCL_1124), KELLY (RRID:CVCL_C885), GI‐ME‐N (RRID:CVCL_1232), SK‐N‐AS (RRID:CVCL1700), SH‐SY5Y (RRID:CVCL_0019), SK‐N‐BE (RRID:CVCL_0529), and SHEP‐2 (RRID:CVCL_0524), were purchased from the DMSZ (German Collection of Microorganisms and Cell Culture GmbH) and SHEP‐21N (RRID: CVCL_9812) were donated by the group of Dr Lin (Baylor College of Medicine). SMS‐KCNR (RRID:CVCL_7134), NB‐1643 (RRID:CVCL_5627), SK‐N‐FI (RRID:CVCL_1702), LAN‐6 (RRID:CVCL_1363), LAN‐5 (RRID:CVCL_0389), and NMB (RRID:CVCL_2143) were obtained from the Children's Oncology Group (St Jude's Hospital, Memphis) with authentication of the cell lines being performed in the form of STR repeat analysis [18].

#### 2.1.2 Prostate cancer cells

LN‐CAP (RRID:CVCL_0385) were purchased from the American Type Culture collection (Rockville, MD, USA).

#### 2.1.3 Cell culture

Cells were routinely cultured at 37 °C and in 5% CO_2_ in Dulbecco's modified Eagle's medium (DMEM) containing 4.5 g·L^−1^ d‐glucose, glutamate and supplemented with 10% (v/v) fetal bovine serum, 2 mm l‐glutamine, 10 U·mL^−1^ penicillin, 10 μg·mL^−1^ streptomycin and MEM non‐essential amino acids (1×). LN‐CAP cells were grown in RPMI 1640 medium containing 10% FBS, penicillin (100 U·mL^−1^), streptomycin (100 μg·mL^−1^), l‐glutamine (2 mm), and sodium pyruvate (1 mm). Cell lines were routinely tested for mycoplasma and were mycoplasma free. The selective PRMT5 inhibitor T1‐44 (Argonaut Therapeutics, Oxford, UK) has been previously described and characterised [19] and the cell lines were incubated for 72 h (8 nm, 40 nm, 200 nm and 1 μm) following which they were processed for the respective assays.

### 2.2 Cell line authentication by short tandem repeat (STR) assays

#### 2.2.1 Neuroblastoma cell lines

All neuroblastoma cell lines have been authenticated within 3 years by short tandem repeat profiling by the ATCC as per the following protocol: briefly, DNA was isolated for all cell lines in sterile conditions and Microsatellite typing was performed with 10 ng of DNA using the PowerPlex 16 system (Promega Corp, Madison, WI, USA) and results were analysed using the genemapper software (Applied Biosystems, Waltham, MA, USA). The following Loci were amplified; Penta E, D18S51, D21S11, TH01, D3S1358, FGA, TPOX, D8S1179, vWA, Amelogenin, Penta D, CSF1P0, D16S539, D7S820, D13S317 and D5S818.

#### 2.2.2 Prostate cancer cell lines

The LN‐CAP cell line was authenticated by short tandem repeat profiling by the ATCC as per the following protocol: briefly, DNA was isolated for LN‐CAP and microsatellite typing was performed with 10 ng of DNA using the PowerPlex 16 system (Promega Corp) and results were analysed using the genemapper software (Applied Biosystems). The following Loci were amplified; Penta E, D18S51, D21S11, TH01, D3S1358, FGA, TPOX, D8S1179, vWA, Amelogenin, Penta D, CSF1P0, D16S539, D7S820, D13S317 and D5S818.

### 2.3 Plasmids and siRNA transfections

Plasmid transfections were carried out for 72 h using the TransFast reagent (Promega) according to the manufacturer's instructions. RNA interference was carried out with 15 nm siRNA using the Oligofectamine transfection reagent (Invitrogen, Waltham, MA, USA), as per the manufacturer's instructions. siRNAs used are as follows: non‐targeting control, 5′‐AGCUGACCCUGAAGUUCUU‐3′, *CPSF3* (human, ID: s28531, Life Technologies, Carlsbad, CA, USA), *CPSF4* (human, ID: s21412, Life Technologies), *HNRNPA3* (human, ID: s47927, Life Technologies), *HNRNPM* (human, ID: s144164, Life Technologies), *SRSF1* (human, ID: s143028, Life Technologies), *SRSF4* (human, ID: s12734, Life Technologies) and *DIABLO* (human, ID:s224426, Life Technologies). Stable GFP‐E2F1 expressing CHP‐134 cell lines were generated by transfecting a pMaX‐GFP vector containing a full‐length *E2F1* insert (plasmid #16007, Addgene, Watertown, MA, USA) into CHP‐134 E2F1 Cr (CRISPR) cells and selecting with G418. Stable E2F1 mutant cell lines were generated by transfecting E2F1 Cr cell lines with pcDNA3.1‐HA E2F1 R113K plasmids expressing the R113K E2F1 mutant [15] and selecting for G418 resistance. Stable DIABLO exon2^+^ and exon2^−^ cell lines were generated by transfecting WT CHP‐134 cell lines with pcDNA3.1‐C‐(k)DYK plasmids expressing the ORF from *Smac*\*DIABLO* transcript NM_019887.6 (OHu27599D: GenScript) or the ORF from *Smac*\*DIABLO* transcript NM_001278303.1 (OHu27556D: GenScript) respectively, followed by selection in G418.

### 2.4 Generation of E2F1 CRISPR/Cas9 knockout cell lines

The following guide RNA sequences were used: forward sgRNA 5′CACCGGAAACTGACCATCAGTACC, and reverse sgRNA: 5′AAACGGTACTGATGGTCAGTTTCC, to generate E2F1 Cr cell lines as described previously [20]. Briefly, sgRNA strands were annealed and phosphorylated using T4 PNK (New England Biolabs, Ipswich, MA, USA) then cloned into the pSpCas9(BB)‐2A‐Puro plasmid (Addgene) cut with *BbsI* (Thermo Fisher, Waltham, MA, USA) and ligated by T4 DNA ligase (New England Biolabs). Plasmids were sequenced to confirm successful incorporation of the sgRNA. CHP‐134 cells were transfected with the plasmid and selected for 48 h with 2 μg·mL^−1^ puromycin. Surviving cells were seeded individually into 96 well plates and colonies were grown out, prior to confirmation of *E2F1* loss by immunoblot. Genomic DNA was extracted (QIAamp DNA Mini Kit, Qiagen, Hilden, Germany) and a small sequence surrounding the Cas9 cleavage site in *E2F1* was PCR amplified prior to sequencing to confirm CRISPR knockout of the *E2F1* gene.

### 2.5 Immunoblots

Cell pellets were washed with PBS and lysed in 1% modified RIPA buffer (50 mm tris–HCl pH 7.5, 150 mm NaCl, 1% Igepal CA‐630 [v/v], 1 mm EDTA, 1 mm NaF, 1 mm Na_3_VO_4_, 1 mm AEBSF, protease inhibitor cocktail) and incubated on ice for 30 min prior to SDS/PAGE and transfer to nitrocellulose. The following antibodies were used in immunoblots: β‐actin (3700S, Cell Signaling Technology, Danvers, MA, USA, dilution 1 : 2000), E2F1 (3742S, Cell Signaling Technology, dilution 1 : 1000), symmetric dimethyl arginine (SDMe) (13222S, Cell Signaling Technology, dilution 1 : 1000), FLAG (clone M2, F1804, Sigma, St. Louis, MO, USA, 1 : 1000), GAPDH (clone 6C5, MAB374, Millipore, Burlington, MA, USA, 1 : 2000), Smac/DIABLO (2954S, Cell Signaling Technology, dilution 1 : 1000), BIM (BCL2L11) (2819, Cell Signaling Technology, dilution 1 : 1000), ACIN1 (A300‐999A, Bethyl, Montgomery, TX, USA, dilution 1 : 1000) CFLAR (AV00022‐QC0240, Sigma‐Aldrich, dilution 1 : 1000), MYCN (SC‐53993, Santa Cruz, Dallas, TX, USA, dilution 1 : 1000), PRMT5 (79998S, Cell Signaling Technology, dilution 1 : 1000), CPSF3 (SC‐393001, Santa Cruz, dilution 1 : 1000), CPSF4 (15023‐I‐AP, ProteinTech, Rosemont, IL, USA, dilution 1 : 1000), SRSF1 (SC‐33652, Santa Cruz, dilution 1 : 1000), SRSF3 (51039S, Cell Signaling Technology, dilution 1 : 1000), SRSF4 (H303‐670A, Bethyl, dilution 1 : 1000), HNRNPA3 (25142‐I‐AP, ProteinTech, dilution 1 : 1000), HNRNPM (TA803154, ORIGENE, Rockville, MD, USA, dilution 1 : 1000).

### 2.6 Cell viability assays

Cells were seeded in 96‐well plates and then maintained in the presence of vehicle or increasing doses of T1‐44 and T1‐68 (0.001 nm–100 μm) for 6 days before the addition of 10 μL of thiazolyl blue tetrazolium bromide (MTT) (Sigma‐Aldrich). Plates were incubated for 4 h and the resultant crystals were dissolved in 1% SDS/0.025 N HCl solution for a further 24 h before absorbance at OD_520_ was measured with a TECAN microplate reader (Tecan, Männedorf, Switzerland). Wells containing medium only were used as background for the measurement. Viability of vehicle control cells was set to 100%. Half maximal effective concentration (IC_50_) values were derived from dose–response curves. IC_50_ values at 144 h were calculated by determining the T1‐44 and T1‐68 concentrations needed to achieve a 50% reduction in cell viability as compared to DMSO‐treated cells (that are set to 100%).

### 2.7 FACS analysis

Neuroblastoma cells (CHP‐134, GI‐ME‐N, SHEP‐2, KELLY and SK‐N‐BE) were harvested for FACS analysis after 144 h treatment with T1‐44 (8 nm–1 μm). Supernatants containing floating cells were collected from the culture dishes and combined with adherent cells collected by treating with 0.05% trypsin/EDTA. Next, cells were washed in PBS prior to fixing with 100% ice‐cold ethanol and staining with 50 μg·mL^−1^ propidium iodide supplemented with 50 μg·mL^−1^ RNAse A in PBS. After 1 h incubation in the dark at room temperature, DNA content of nuclei (cell cycle distribution and subG1) was analysed using a BD Accuri C6 flow cytometer with cflow plus software or flowjo software (BD Biosciences, Franklin Lakes, NJ, USA). A total of 20 000 events were collected per sample.

### 2.8 RNA isolation and quantitative RT‐PCR

RNA was isolated from cells using TRIzol (Thermo Fisher Scientific) according to the manufacturer's instructions. 1 μg of total RNA and oligo (dT)20 (Invitrogen) was used for complementary DNA (cDNA) synthesis using SuperScript III Reverse Transcriptase (Invitrogen) as per the manufacturer's instructions. Quantitative PCR (qPCR) was then carried out in technical triplicate using the indicated primer pairs and the Brilliant III SYBR Green qPCR Master Mix (Stratagene, San Diego, CA, USA) on an AriaMx (Agilent, Santa Clara, CA, USA) instrument. Results were expressed as average relative expression using the Δ*C*_t_ method from at least three biological repeat samples. Glyceraldehyde‐phosphate dehydrogenase (*GAPDH*) primer sets were used as the internal calibrator. For splicing analysis, primers spanning exon junctions were designed and the signal of exon inclusion forms and exon exclusion forms of a transcript were first normalised to *GAPDH* (Δ*C*_t_). The inclusion/exclusion ratio was then calculated using the formula $2^{-\left[\Delta C_{t}\;\text{inclusion}-\Delta C_{t}\;\text{exclusion}\right]}$. Error bars represent SD unless otherwise indicated. For primer lists, please see Table S1.

### 2.9 RNA sequencing

WT E2F1 CHP‐134, E2F1 Cr CHP‐134, and GI‐ME‐N cells were treated with 200 nm concentration of PRMT5 inhibitor (T1‐44) or DMSO as a negative control, for 72 h. Total RNA (from biological triplicates) was isolated using TRIzol (Thermo Fisher Scientific) according to the manufacturer's instructions. RNA‐sequencing was performed by BGI Genomics. Briefly, an Agilent 2100 Bioanalyzer (Agilent RNA 6000 Nano Kit) was used for RNA sample quality control purposes (RNA concentration, RIN value, 28S/18S, and the fragment length distribution). mRNAs were isolated from total RNA using oligo(dT)‐based mRNA enrichment. mRNA was fragmented and reverse transcribed with random N6 primer, prior to second strand synthesis, end repair and A‐tailing. This was followed by bubble adaptor ligation, PCR amplification and heat treatment to get single‐stranded DNA. This was circularised for DNBseq (PE150 at ≥ 30 million reads per sample coverage). Gene expression data have been deposited in the National Center for Biotechnology Information's (NCBI) Gene expression Omnibus (GEO) and are accessible through GEO Series accession number GSE243989.

### 2.10 RNA‐seq analysis

RNA‐seq raw data was processed with fastp software [21] to remove sequencing adaptors and low‐quality bases. The processed reads were aligned with human reference genome hg19 using the star package (v.020201) [22] with –quatMode Genecounts option to calculate the read counts. The differential expression analysis was performed with deseq2 r package [23]. Genes were considered differentially expressed (DEGs) if the adjusted *P*‐value, calculated using the Benjamini–Hochberg method in order to minimise the false discovery rate, was less than 0.01. We further filtered significant DEGs with fold change cut‐offs, usually set to a 2‐fold change in absolute expression level (equivalent to a log2 fold change of 1), unless otherwise indicated. The aligned data was also used for differential alternative splicing assessment with the rmats program (v4.0.2) [24]. The FDR threshold for differential per cent spliced in (PSI) values was chosen to be 0.01.

### 2.11 Gene set enrichment analysis

Genes significantly differentially expressed upon T1‐44 treatment were further subjected to pathway enrichment analysis using the pathfindr [25], and topgo [26], software packages to reveal signalling and metabolic pathways, as well as GO categories, over‐represented in the DEG sets. For pathway‐based gene enrichment analyses with pathfindr, we used KEGG [27] genesets and IntAct [28] molecular interaction resource data as active subnetworks. *P*‐values obtained from the enrichment tests were adjusted (Bonferroni method) and significantly enriched pathways were identified using a threshold of 0.05 imposed on the adjusted *P*‐value. The GO:BP enrichment analysis in differentially spliced gene sets was performed using the R Bioconductor topgo r package (v.2.52.0) using weight01 algorithm [29].

### 2.12 Chromatin immunoprecipitation

ChIP samples were prepared as described previously [30], using 3 μg of appropriate antibody (control rabbit IgG, anti‐E2F1 [A300‐766A], Bethyl Laboratories, anti‐MYCN [ab227822, Abcam, Cambridge, UK]) and pre‐blocked protein A beads. DNA was isolated by using the QIAquick PCR purification kit according to the manufacturer's instructions. The purified DNA was prepared for quantitative real‐time PCR using SYBR FAST qPCR Kit (Agilent). Experiments were performed in triplicates on an AriaMx Real‐Time PCR System (Agilent Technologies). The *PARP2* (binding site 2; P2BS2) and *CDC6* promoters were used as positive controls for MYCN and E2F1 binding respectively. The primer sequences used in qPCR are given in Table S1.

### 2.13 Gene expression and bioinformatics analysis

The gene expression and clinical data from 649, 579, 498 and 248 neuroblastoma patients were downloaded from GEO (GSE45547, GSE73517, GSE3960 and GSE62564 RNA‐seq data series). The microarray data from neuroblastoma patient cohorts were downloaded from the R2 database [R2: Genomics analysis and visualisation platform (http://r2.amc.nl)]. The relative gene expression of *PRMT5*, *E2F1* and *MYCN* and survival analysis were generated using the 44 k oligonucleotide microarrays. Kaplan Meier survival curves (overall and event‐free survival of the patients in each dataset) were obtained based on single colour probes that showed a present or absent call on a particular gene from *n* = 476 and *n* = 498 tumours that showed a present call for the expression of the gene of interest and for which survival data of up to 216 months was available for the patients. A multivariate Cox analysis was used to analyse the prognostic significance of each gene of interest, with gene expression values used to generate two groups of samples (high versus low expression denoted in blue and red respectively). For the correlational analysis of the genes of interest (*PRMT5*, *E2F1*, and *MYCN*) with neuroblastoma stage, a total of 571 and 441 stage 1–4 neuroblastomas were analysed and *P*‐values were calculated with a Wilcoxon rank sum test for box‐plots. Stage 4s (*n* = 78 and *n* = 52 respectively) tumours were excluded from this analysis. For the correlational analysis of *PRMT5*, *E2F1* and *MYCN* with the *MYCN* amplification status of the patients, a total of 643 and 493 neuroblastoma patients subdivided into *MYCN* amplified and non‐amplified tumours was analysed and *P*‐values were calculated with a Wilcoxon rank sum test for box‐plots. Neuroblastomas, wherein *MYCN* amplification status was undefined (*n* = 6 and *n* = 5), were excluded from this analysis. Neuroblastoma patient datasets from TARGET were accessed using ucsc xena Browser (https://xena.ucsc.edu) [31], and the TARGET Pan‐Cancer (PANCAN) study. Expression data for *E2F1*, *MYCN*, *PRMT5* and a number of splicing factors were downloaded from 162 patients (as RNA‐seq by expectation‐maximisation [RSEM] TPM values). Exon coordinates output from the rMATS package used for splicing analysis were utilised to identify ENSEMBL transcripts of interest (containing or lacking the skipped exon with appropriate flanking exons) derived from apoptotic genes. When available, the expression data in patient samples for these transcripts were also downloaded (as RSEM isoform percentage values). If a transcript represented only a small fraction of all transcripts from the gene (0–5%) it was discarded. Data for ENST00000309955 (*CFLAR* transcript containing exon 2) and ENST00000338631 (*ACIN1* transcript excluding exon 2) were downloaded, though transcripts of interest derived from *BCL2L11* and *DIABLO* were either not present in the patient dataset or did not meet the required criteria. Patient samples were ranked into quartiles based on a primary criterion (i.e. *E2F1* expression) and expression of other variables (i.e. isoform percentage values) was displayed graphically using graphpad software (DotMatics, Boston, MA, USA).

### 2.14 Statistical analysis

All quantitative data points represent the mean from three independent biological experiments with standard deviation (SD). Unless indicated in the figure legend, statistical analysis was performed using one‐way ANOVA with Tukey's multiple comparison test or unpaired *t*‐tests (graphpad Software). The IC50 value was determined by nonlinear regression (curve fit) using the log (inhibitor) versus response (three parameters) model.

## 3 Results

### 3.1 MYCN, PRMT5 and E2F1 expression in NB cell lines

**Fig. 1:**
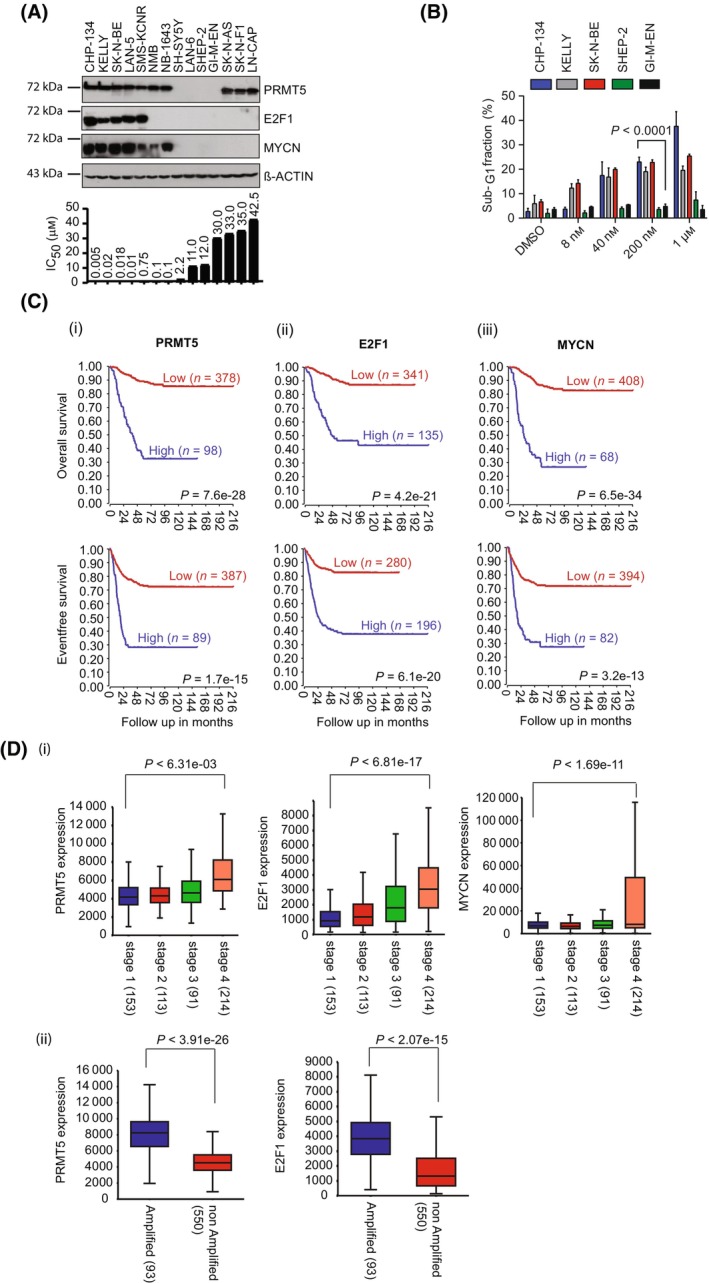
High expression of PRMT5, E2F1 and MYCN correlates with poor prognosis in neuroblastoma and sensitivity to a PRMT5‐specific inhibitor (T1‐44). (A) A representative Immunoblot (*n* = 3) displaying protein levels of PRMT5, E2F1 and MYCN in a panel of neuroblastoma cell lines grouped according to their E2F1 and MYCN expression levels. LN‐CAP prostate cancer cells were included as a control cell line without *MYCN* amplification. IC_50_ values representing sensitivity of each cell line to PRMT5 inhibitor T1‐44 are displayed at the bottom of the immunoblot. (B) FACs analysis of three sensitive cell lines and two insensitive cell lines measuring the Sub‐G_1_ fraction of cells stained with propidium iodide following treatment with increasing concentrations of T1‐44 (8 nm–1 μm) for 144 h. Results are the mean values ± SD; One‐way ANOVA with Tukey's multiple comparison test performed between CHP‐134 and GI‐ME‐N cells at 200 nm concentration: adjusted *P* < 0.0001; *n* = 3 independent experiments (each with three technical replicates). (C) Overall (above) and event‐free (below) patient survival probability in stage 1‐stage 4 neuroblastoma patients in the Kocak databases (*n* = 634 patients with survival data available out of the cohort of 649) with respect to the *PRMT5*, *E2F1* and *MYCN* mRNA expression in these tumours. *P* values were calculated with a log‐rank test for survival curves. (D) (i) Correlation of *PRMT5* (*P* < 6.31e‐03 between stage 1 and stage 4 neuroblastoma), *E2F1* (*P* < 6.81e‐17 between stage 1 and stage 4 neuroblastoma), *MYCN* (*P* < 1.69e‐11 between stage 1 and stage 4), and INSS (International Neuroblastoma Staging System) neuroblastoma stage 1 (*n* = 153), stage 2 (113), stage 3 (*n* = 93) and stage 4 (*n* = 214). Kruskal–Wallis with Dunn's multiple comparisons test was used to determine *P* values, which were corrected with the Bonferonni correction method. (ii) Box plot correlational analysis of *PRMT5* (*P* < 3.98e‐26) and *E2F1* (*P* < 2.07e‐15) mRNA in the *MYCN* amplified (*n* = 550) and non‐*MYCN* amplified (*n* = 93) neuroblastoma tumours. *P* values were calculated with a two‐sided Wilcoxon rank sum test for box‐plots. Box‐plot centre represents mean, the box represents SD, and whiskers represent minimum and maximum.

Neuroblastoma cell lines are generally reflective of the disease from which they were derived and occur in two broad phenotypes; epithelial‐like which exhibits neuronal features, or the mesenchymal phenotypes with non‐neuronal features [32, 33]. We examined a collection of 13 neuroblastoma cell lines (Fig. 1A) for levels of PRMT5, E2F1 and MYCN which subsequently allowed us to broadly divide them into high PRMT5, E2F1 and MYCN expressors (CHP‐134, KELLY, SK‐N‐BE, LAN‐5 and SMS‐KCNR cells) or low expressors of PRMT5, E2F1 and MYCN (GI‐ME‐N, SHEP‐2, SH‐SY5Y and LAN‐6 cells). Other cell lines in the panel showed significant expression of PRMT5 and MYCN (NMB and NB‐1643 cells) or PRMT5 only (SK‐N‐AS and SK‐N‐FI cells); an analysis of the RNA expression exhibited a similar trend and coincided with protein levels (Table S2). We tested the role of PRMT5 using a selective inhibitor, T1‐44 [19]. We evaluated cell viability upon treating with T1‐44 and observed a range of sensitivities across the cell lines, where cells expressing high MYCN, E2F1 and PRMT5 were, generally, more sensitive to T1‐44 treatment (CHP‐134 IC_50_ of 5 nm) than cells expressing lower levels (GI‐ME‐N IC_50_ 30 μm) these results were confirmed with another selective PRMT5 inhibitor GSK591 (which though less potent than T1‐44, showed a similar trend in terms of the sensitivity of the cells with the compound) [34] (Fig. S1A; Table S2). The level of apoptosis (sub‐G1 cells) was similarly most apparent in NB cells with high MYCN, PRMT5 and E2F1 (Fig. 1A,B and Fig. S1B). The symmetric dimethyl arginine (SDMe) mark was reduced upon T1‐44 treatment in all the cell lines (Fig. S1A). We conclude from the analysis performed here that high PRMT5, E2F1 and MYCN confers sensitivity to PRMT5 inhibition resulting in cell death in the cell lines studied.

### 3.2 *PRMT5* and *E2F1* in clinical disease

To address whether there was any potential clinical significance in the levels of *PRMT5* and *E2F1*, we examined gene expression in a collection of 649 human neuroblastoma biopsies taken from early and late (stage 1 to 4) disease and calculated survival probability curves [35]. High *PRMT5* and *E2F1* expression correlated with poor overall and event‐free survival relative to patients with low *PRMT5* and *E2F1* expression (Fig. 1C), and *PRMT5* and *E2F1* expression was highest in stage 4 compared to stage 1, 2 and 3 disease (Fig. 1D). The expression of *MYCN*, an established prognostic biomarker for high‐risk neuroblastoma [9] was similarly correlated with poor overall survival and expressed at high levels in late‐stage disease (Fig. 1C,D). Further, there was a highly significant correlation between an amplified *MYCN* gene, and levels of *PRMT5* and *E2F1* gene expression in the tumour tissue (Fig. 1D). These findings were corroborated across another independently verified and annotated set of neuroblastoma biopsies (SEQC; *n* = 498 patients) [36] where in a similar way to the other biopsy collection, high *PRMT5* and *E2F1* coincided with *MYCN* expression, occurred in stage 4 disease and correlated with poor overall survival (Fig. S1C,D).

### 3.3 PRMT5 controls alternative RNA splicing

To gather mechanistic information on the role of PRMT5 and E2F1 in NB, we chose to focus our experiments on the two cell lines which represent the extreme of each expression condition: CHP‐134, with high expression levels of MYCN, PRMT5 and E2F1, and GI‐ME‐N with correspondingly lower expression levels (Fig. 1A,B), and where the effect of PRMT5 inhibition on cell viability exhibited a 6000‐fold difference (5 nm compared to 30 μm IC50, respectively; Table S2).

**Fig. 2:**
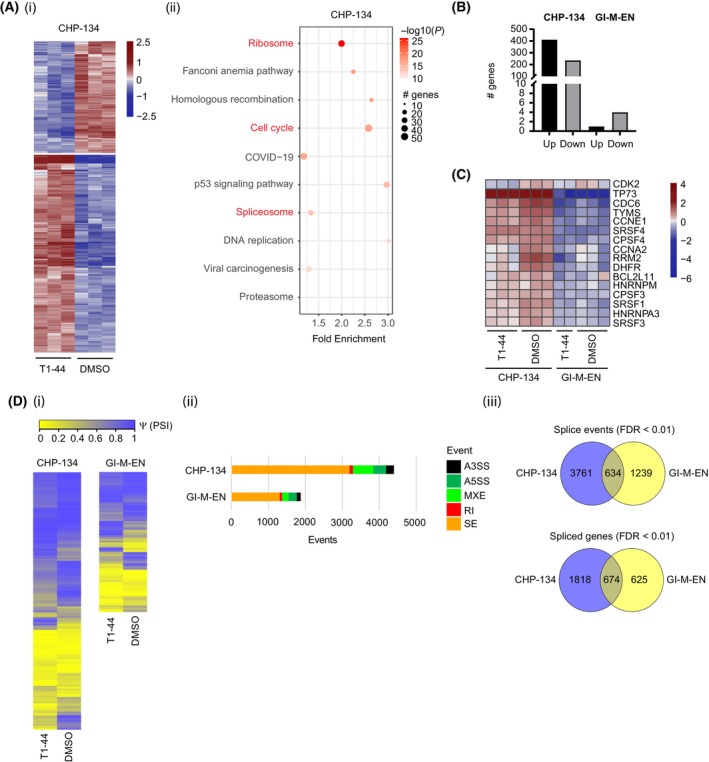
PRMT5 inhibition by T1‐44 deregulates alternative splicing events in sensitive cell lines. (A) (i) A heat map displaying significant differences in the differentially expressed genes (DEGs) between T1‐44 treated and DMSO control treated CHP‐134 cells. Normalised variance stabilising transformation (vst)‐transformed gene expression values corresponding to significantly differentially expressed genes (FDR < 0.01, log2(FC) > 1) were mean‐centered by rows. Each row of the heatmap represents transformed expression values of one differentially expressed gene (DEG) across all samples (blue: low expression; red: high expression). These data were generated from three independent biological samples (*n* = 3). (ii) Functional characterisation of CHP‐134 differentially expressed genes using pathfindR. KEGG terms related to cell cycle, ribosome and spliceosome are highlighted in red (https://www.genome.jp/kegg‐bin/show_pathway?ko03040). (B) Distinct differential gene expression changes between T1‐44 treated and DMSO control treated CHP‐134 (E2F1 and MYCN high) and GI‐ME‐N (E2F1 and MYCN low) cell lines are represented in graphical form as a count for upregulated and downregulated genes (*P*adj < 0.01, log2FC > 1) (*n* = 3). (C) A heatmap displaying the normalised variance stabilising transformation (vst)‐transformed expression values from each biological replicate of T1‐44 or DMSO‐treated CHP‐134 and GI‐ME‐N cells used in the RNA‐seq analysis for 16 E2F1 target genes of interest (selected from RNA splicing factors and classical E2F1 target genes involved in cell cycle progression) (*n* = 3). (D) (i) Differential changes in splicing between T1‐44 treated and DMSO control treated CHP‐134 and GI‐ME‐N cell lines are displayed as a heatmap of PSI values (Ψ, per cent spliced in) for all significant differential splicing events (FDR < 0.01). These data were generated from three independent biological samples. (ii) The bar chart displays the statistically significant differential splicing events in each cell line after treatment with T1‐44. The total number of these splicing changes corresponding to different types of splicing event is displayed in different colours. A3SS, alternative 3′ splice; A5SS, alternative 5′ splice site; MXE, mutually exclusive exons; RI, retained intron; SE, skipped/cassette exon. (iii) Venn diagrams showing the overlap between differential splicing events or differentially spliced genes identified in each of the cell lines after T1‐44 treatment.

To gain insight into the role of PRMT5 and E2F1, we first performed RNA‐seq on CHP‐134 and GI‐ME‐N cells with or without treatment (T1‐44 at 200 nm). A genome‐wide analysis showed that differential gene expression was far more evident in CHP‐134 cells compared to GI‐ME‐N cells upon T1‐44 treatment (Fig. 2A(i),B and Table S3). In the CHP‐134 cells, about 647 genes were identified to be differentially expressed genes (DEGs: *P*adj < 0.01, log_2_(FC) > 1) upon treatment with 412 increased and 235 decreased (Fig. 2B). Far less differential expression was apparent in GI‐ME‐N cells, with only 4 down‐ and 1 upregulated genes were evident under the same treatment conditions and cut‐off criteria (Fig. 2B). Both KEGG pathway and gene ontology (GO) analyses performed on genes significantly differentially expressed in CHP‐134 cells revealed enrichment in pathways and categories associated with cell cycle, apoptosis, ribosome and spliceosome (Fig. 2A (ii) and Fig. S2A). The enrichment was especially pronounced upon PRMT5 inhibition (Fig. 2A (ii) and Fig. S2A), suggesting that PRMT5 plays a pleiotropic role in CHP‐134 cells, which includes pathways that affect gene expression and RNA splicing. Further and consistent with the high level of E2F1 in CHP‐134 cells, many E2F target genes (identified from mining the RNA‐seq data sets) were highly expressed in CHP‐134 but much lower in GI‐ME‐N cells (Fig. 2C).

The enrichment of the KEGG spliceosome pathway category (Fig. 2A (ii)) prompted us to examine the level of RNA splicing in each cell type. Using the rMATS algorithm on the RNA‐seq data set [24], alternative RNA splicing was found to be prevalent in CHP‐134 cells with a highly significant (FDR < 0.01) quantitative impact on the splicing events upon treatment with T1‐44 (Fig. 2D, Table S4). Affected splicing events included skipped exons, retained introns, alternative 3′ (A3SS) or 5′ (A5SS) splice sites and mutually excluded exons (Fig. 2D (ii)). In CHP‐134 cells, over 2400 genes had the splicing pattern significantly affected (FDR < 0.01) upon PRMT5 inhibition, compared to GI‐ME‐N where about 1300 genes were affected (Fig. 2D (iii)). There was some overlap, but equally major differences were apparent between the two NB cell lines in the genes subjected to alternative splicing, highlighting differences in the RNA splicing programme between the two cell types (Fig. 2D (iii)). An analysis of the GO term enrichment in differentially spliced genes in the CHP‐134 cells highlighted telomere maintenance and various aspects of the mitotic cell cycle, in addition to terms connected with the regulation of apoptotic processes (Fig. S2B,C).

### 3.4 PRMT5 and E2F1 foster alternative RNA splicing

**Fig. 3:**
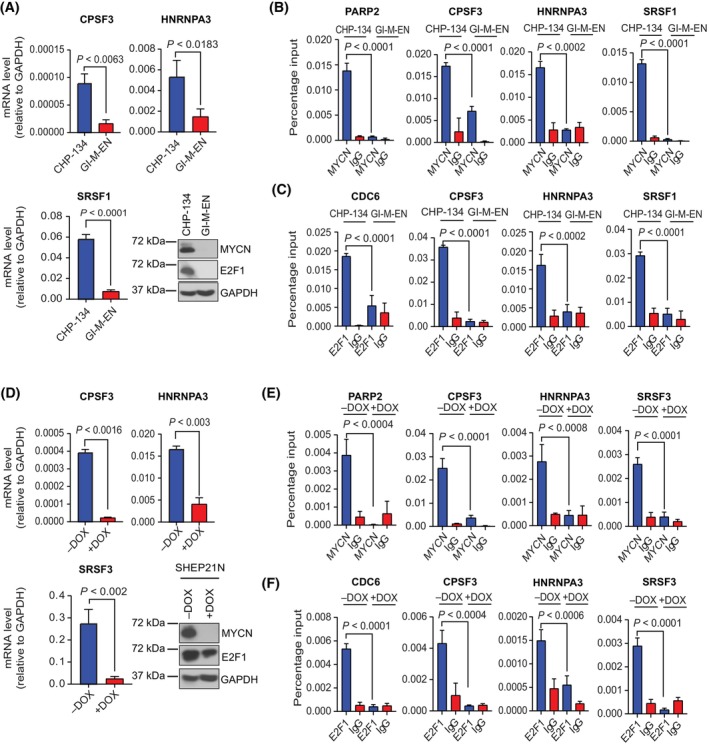
Splicing factors are transcriptional targets for E2F1 and MYCN in neuroblastoma cell lines. (A) The mRNA expression level of splicing factors *CPSF3*, *SRSF1* and *HNRNPA3* in CHP‐134 and GI‐ME‐N cell lines. Significance was calculated with an unpaired *t*‐test. Results are the mean values ± SD; *n* = 3 independent experiments (each with three technical replicates). Immunoblot showing the protein expression levels of MYCN and E2F1 in the CHP‐134 and GI‐ME‐N cell lines. GAPDH served as a loading control for this experiment. (B, C) ChIP assays denoting the binding affinity of MYCN and the E2F1 transcription factors on the promoter region of the splicing factor genes *CPSF3*, *HNRNPA3* and *SRSF1* in both CHP‐134 and GI‐ME‐N cell lines. Results represent mean percentage enrichment values ± SD; Significance was calculated with one‐way ANOVA with Tukey's multiple comparison test; (*n* = 3 independent experiments, each with three technical replicates). *PARP2* and *CDC6* served as a positive control for the binding of MYCN and E2F1 respectively. (D) The mRNA expression levels of splicing factors *CPSF3*, *HNRNPA3* and *SRSF3* in a MYCN SHEP‐21N Tet‐off inducible cell line treated with (+DOX) or without (−DOX) doxycycline; Significance was calculated with an unpaired *t*‐test. Results represent mean expression values ± SD; *n* = 3 independent experiments (each with three technical replicates). A Representative immunoblot (*n* = 3 independent experiments) showing the protein expression levels of MYCN and E2F1 in SHEP‐21N (−DOX) MYCN overexpressing cells and the SHEP‐21N (+DOX) non‐MYCN‐expressing cells following treatment with doxycycline for 72 h. GAPDH served as a loading control for this experiment. (E) Chromatin immunoprecipitation (ChIP) assays denoting the binding affinity of the MYCN transcription factor on the promoter region of the splicing factor genes *CPSF3*, *HNRNPA3* and *SRSF3* in both SHEP‐21N cells overexpressing MYCN (−DOX) and in cells with decreased MYCN expression (+DOX for 72 h). Results represent mean percentage enrichment values ± SD; Significance was calculated with a one‐way ANOVA with Tukey's multiple comparison test; *n* = 3 independent experiments (each with three technical replicates). *PARP2* served as a positive control for the binding of MYCN. (F) Chromatin immunoprecipitation (ChIP) assays denoting the binding affinity of the E2F1 transcription factor on the promoter region of the splicing factor genes *CPSF3*, *HNRNPA3* and *SRSF3* in both SHEP‐21N cells overexpressing MYCN (−DOX) and in cells with decreased MYCN expression (+DOX for 72 h). Results represent mean percentage enrichment values ± SD; Significance was calculated with a one‐way ANOVA with Tukey's multiple comparison test; *n* = 3 independent experiments (each with three technical replicates). *CDC6* served as a positive control for the binding of E2F1.

Alternative RNA splicing is a regulated process that requires a diverse group of splicing and RNA processing proteins [37]. Therefore, to determine whether the differences seen in alternative RNA splicing reflected altered expression of splicing factors and RNA processing genes, we examined differentially expressed splicing factors between CHP‐134 and the GI‐ME‐N cell lines; *CPSF3* and *4* (polyadenylation specificity factor subunits), *HNRNPA3* and *HNRNPM* (heterogeneous nuclear ribonucleoprotein) and *SRSF1*, *3* and *4* (serine and arginine rich splicing factor) [38]. In the RNA‐seq data set, a significant difference in RNA level of *CPSF3‐4*, *HNRNPM*, *HNRNPA3*, *SRSF1*, *3* and *4* between the CHP‐134 and GI‐ME‐N cells was apparent, where CHP‐134 was the highest expressor (Figs 2C and 3A and Fig. S3A). These results were confirmed in another cell line, KELLY, also representing high MYCN, PRMT5 and E2F1 expressing cells which exhibited higher expression of the splicing factors than the other cell line, SHEP‐2, with low expression of MYCN, PRMT5 and E2F1 (Fig. S4A). We then tested whether the genes were direct transcription targets for MYCN and E2F1 through chromatin‐immuno‐precipitation (ChIP) assays. We found that all the genes tested were significant chromatin targets for both MYCN and E2F1 in CHP‐134 and KELLY cells, with little ChIP activity detectable in GI‐ME‐N or SHEP‐2 cells (Fig. 3B,C and Figs S3B,C and S4C).

We confirmed these results using a number of other experimental tools, which included an MYCN inducible Tet‐off SHEP‐2 cell line, where ectopic MYCN expression from a tetracycline‐regulated gene was only apparent under Tet‐off (−DOX treatment) conditions (Fig. 3D and Fig. S3D) [39]. Upon MYCN expression, there was also a notable increase in endogenous E2F1 levels (Fig. 3D), an observation consistent with previous reports that the *E2F1* gene is a target for MYC proteins [40, 41]. Most of the splicing factor and RNA processing protein genes were ChIP targets for both MYCN and E2F1 (Fig. 3E,F and Fig. S3E,F) and were similarly induced upon MYCN expression (Fig. 3D, Figs S3D and S4G). These results show that the splicing factor and RNA processing protein genes identified in the RNA‐seq data which are highly expressed in CHP‐134 are transcription targets for MYCN and E2F1.

### 3.5 Alternative RNA splicing and apoptosis

**Fig. 4:**
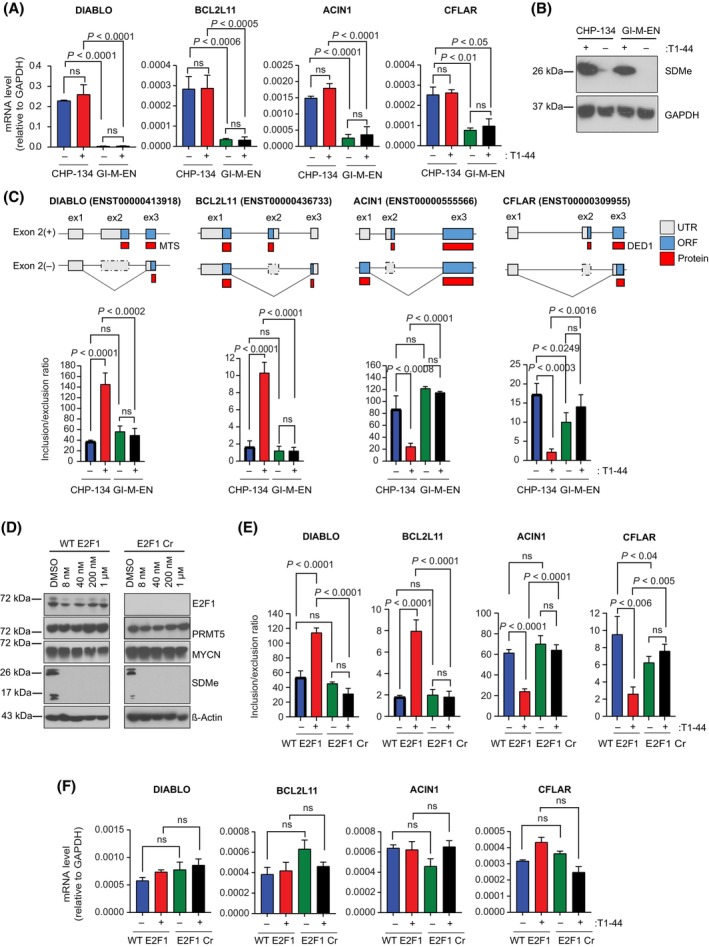
Changes in the differential splicing events of the apoptotic genes, *DIABLO*, *BCL2L11*, *ACIN1*, and *CFLAR* upon treatment with T1‐44 in CHP‐134. (A) mRNA expression levels of *DIABLO*, *BCL2L11*, *ACIN1* and *CFLAR* in CHP‐134 and GI‐M‐EN cells treated with T1‐44 (200 nm) or DMSO for 72 h. Results represent the mean expression values ± SD; significance was calculated with a one‐way ANOVA with Tukey's multiple comparison test; *n* = 3 independent experiments (each with three technical replicates). (B) A representative Immunoblot (*n* = 3 independent experiments) displaying symmetric dimethylation (SDMe) protein levels in CHP‐134 and GI‐ME‐N cells treated with and without T1‐44 (200 nm) for 72 h. GAPDH served as a loading control for these experiments. (C) Changes in the differential splicing events of the apoptotic genes *DIABLO*, *BCL2L11*, *ACIN1*, and *CFLAR* upon treatment of CHP‐134 and GI‐ME‐N cell lines with T1‐44 for 72 h. Results represent mean inclusion/exclusion ratios of the skipped exons ± SD; significance was calculated with a one‐way ANOVA with Tukey's multiple comparison test; *n* = 3 independent experiments (each with three technical replicates). Above each graph is a schematic representation of the differential splice events in each gene. Exon structure for the skipped exon and flanking exons is displayed, with untranslated regions (UTR) and the open reading frame (ORF) marked in grey and blue respectively. The predicted impact of each splicing event on the ORF and derived protein sequence (displayed in red) are also included. If the skipped exon is known to encode for an amino acid sequence contributing to an annotated protein domain, this is also indicated. DED1, Death effector domain 1; MTS, mitochondrial targeting signal. (D) A representative Immunoblot (*n* = 3 independent experiments) displaying the protein levels of E2F1, PRMT5, MYCN and symmetric dimethylation (SDMe) in wild‐type E2F1 (WT E2F1) and CRISPR E2F1 knockout (E2F1 Cr) CHP‐134 cell lines treated for 144 h with four increasing T1‐44 concentrations (8 nm, 40 nm, 200 nm and 1 μm). ß‐actin served as a loading control and SDMe served as a control for PRMT5 activity. (E) Changes in the differential splicing of the apoptotic genes *DIABLO*, *BCL2L11*, *ACIN1* and *CFLAR* upon treatment of wild‐type (WT) E2F1 and E2F1 CRISPR (Cr) CHP‐134 cell lines with T1‐44 for 72 h. Results represent the mean inclusion/exclusion ratios of the skipped exons ± SD; significance was calculated with a one‐way ANOVA with Tukey's multiple comparison test; *n* = 3 independent experiments (each with three technical replicates). (F) mRNA expression levels of *DIABLO*, *BCL2L11*, *ACIN1* and *CFLAR* in wild‐type (WT) E2F1 and E2F1 CRISPR (Cr) CHP‐134 cells treated with T1‐44 or DMSO for 72 h. Results represent the mean expression values ± SD; significance was calculated with a one‐way ANOVA with Tukey's multiple comparison test; *n* = 3 independent experiments (each with three technical replicates).

The inhibition of PRMT5 led to higher levels of apoptosis in CHP‐134 cells which, at the level of gene expression, coincided with a genome‐wide change in transcription and alternative RNA splicing (Fig. 2). The enriched apoptotic terms, seen in the GO analysis of the rMATS data (Fig. S2C), subsequently led us to test if any genes functionally connected with apoptosis were subjected to an alternative RNA splicing event that impacted their biological activity. We focused on DIABLO, BIM (BCL2L11), ACIN1 and CFLAR, as they are proteins which are central to mediating apoptosis [42, 43, 44, 45, 46, 47]. The steady‐state RNA expression (by qPCR) established that CHP‐134 cells express higher levels than GI‐ME‐N, and the effect of T1‐44 treatment on the steady‐state RNA level was minimal (Fig. 4A). When we further examined the RNA species present in CHP‐134 cells, we identified an RNA variant derived from these genes that had an alternative splicing event around an exon that had potential to influence the biological activity of the protein product (Fig. 4C and Fig. S5A) [48, 49]. For example, the alternative splicing event identified in *DIABLO* disrupted part of the protein‐coding region for the N‐terminal mitochondrial targeting sequence [50] and similarly, the alternative splicing event identified in *CFLAR* had the potential to impact one of the death effector domains encoded by the protein [48] (Fig. 4C).

We further noted that in CHP‐134 cells, MYCN was detected at the promoters of each of the four apoptotic target genes, whilst there was no MYCN ChIP signal detected in the GI‐ME‐N cell line (Fig. S4D) and, at the protein level, CHP‐134 showed higher protein expression of these genes than the GI‐ME‐N cell line (Fig. S4E). The observation was substantiated using the SHEP‐2 MYCN Tet‐off cell line, where MYCN was recruited to *DIABLO*, *BCL2L11* and *ACIN1* promoters under the −Dox condition (Fig. S4F). It is consistent with the high RNA expression and MYCN ChIP activity that the apoptotic proteins were detected at higher levels in CHP‐134 (Fig. S4E (ii)), and furthermore, under the induced MYCN expression conditions in SHEP‐2 cells, increased levels of the apoptotic proteins ACIN1, BCL2L11 and DIABLO was evident (Fig. S4G (ii)).

### 3.6 PRMT5 and E2F1 influence alternative splicing of apoptotic genes

Subsequently, we established an assay to measure the impact of PRMT5 and E2F1 on the splicing of RNA derived from apoptotic genes by measuring the inclusion/exclusion ratio (a measure of exon skipping) of the relevant exonic RNA sequence in each gene. By RT‐qPCR we measured the relative expression of RNA variants that contain the exon of interest, as compared to the expression of RNA variants that lack the exon [51]. Alternative splicing of exon 2 was apparent in the RNA of *DIABLO* and *BCL2L11* in CHP‐134 cells, where the level of exon 2 inclusion increased (and therefore exon skipping decreased) upon treatment with T1‐44 (Fig. 4C). This effect on splicing was not apparent in GI‐ME‐N cells, where splicing of the same exon was not affected by T1‐44 treatment (Fig. 4C). The opposite effect on exon inclusion was apparent in *ACIN1* and *CFLAR* RNA in CHP‐134 cells, where T1‐44 treatment decreased the level of exon 2 inclusion, and therefore exon skipping increased (Fig. 4C). Thus, PRMT5 regulates alternative RNA splicing in genes that mediate apoptosis.

**Fig. 5:**
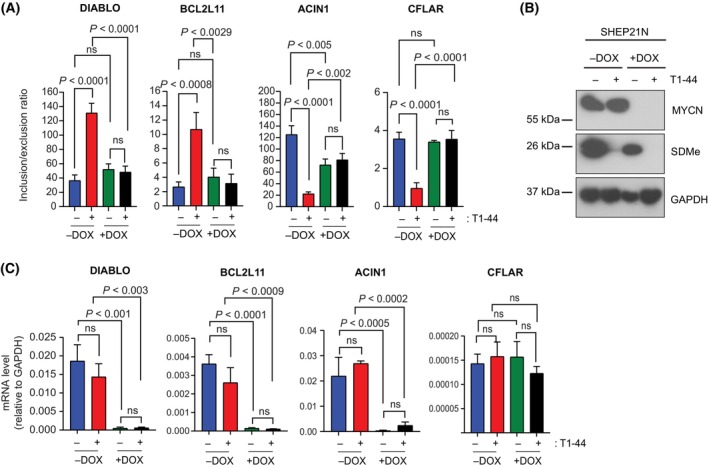
Changes in the differential splicing events of the apoptotic genes, *DIABLO*, *BCL2L11*, *ACIN1*, and *CFLAR* upon treatment with T1‐44 in SHEP‐21N upon MYCN induction. (A) Changes in the differential splicing of the apoptotic genes *DIABLO*, *BCL2L11*, *ACIN1*, and *CFLAR* upon the treatment of SHEP‐21N Tet‐off cells expressing MYCN (−DOX) and SHEP‐21N cells not expressing MYCN (+DOX) with T1‐44 for 72 h. Results are the mean inclusion/exclusion ratios of the skipped exons ± SD; significance was calculated with a one‐way ANOVA with Tukey's multiple comparison test; *n* = 3 independent experiments (each with three technical replicates). (B) A representative Immunoblot (*n* = 3 independent experiments) displaying symmetric dimethylation (SDMe) protein levels in SHEP‐21N cells expressing MYCN (−DOX) and SHEP‐21N cells expressing no MYCN (+DOX) treated with T1‐44 (200 nm) or DMSO for 72 h. GAPDH served as a loading control for these experiments. (C) mRNA expression levels of *DIABLO*, *BCL2L11*, *ACIN1* and *CFLAR* in SHEP‐21N cells overexpressing MYCN (−DOX) and SHEP‐21N cells without MYCN (+DOX) treated with T1‐44 or DMSO for 72 h. Results represent the mean expression values ± SD; significance was calculated with a one‐way ANOVA with Tukey's multiple comparison test; *n* = 3 independent experiments (each with three technical replicates).

We progressed on to evaluate whether E2F1 similarly had a role in the exon skipping events. For this purpose, we first prepared a CHP‐134 cell line in which the *E2F1* gene had been inactivated by CRISPR, described here as CHP‐134 E2F1 Cr (Fig. 4D and Fig. S5B, which represent two different E2F1 Cr cell lines). We compared WT E2F1 cells to the E2F1 Cr cells for any impact on alternative RNA splicing of the apoptotic genes. Remarkably, the exon skipping event affected by T1‐44 treatment was completely lost in the E2F1 Cr cells relative to the WT E2F1 cells (Fig. 4E and Fig. S5C), whilst there was little effect on the steady‐state RNA level derived from each gene (Fig. 4F). We also tested the effect of MYCN using the inducible cells (Fig. 5A). When MYCN expression was induced, the exon2 inclusion event in *DIABLO* and *BCL2L11* RNA increased upon T1‐44 treatment whereas *CFLAR* and *ACIN1* behaved in the opposite way, where decreased exon 2 inclusion was evident (Fig. 5A,B), and the overall steady‐state expression of the gene in these conditions remained similar (Fig. 5C).

We established that the same alternative splicing events occurred in other cell lines that differed in the level of MYCN, PRMT5 and E2F1 expression, namely KELLY and SHEP‐2 (high PRMT5, E2F1 and MYCN and low PRMT5, E2F1 and MYCN respectively; Fig. 1A and Fig. S1A). We found that KELLY cells exhibit increased exon inclusion in *DIABLO* and *BCL2L11* upon T1‐44 treatment, whilst exon inclusion decreased in *ACIN1* and *CFLAR* RNA (Fig. S5F) in a similar way to what was observed for CHP‐134 cells (Fig. 4C). SHEP‐2 cells underwent little change in splicing activity upon T1‐44 treatment (Fig. S5F), and in this respect resembled the splicing events observed in GI‐ME‐N cells (Fig. 4C). Again, there was no significant change in total RNA expression of any of the genes in conditions of T1‐44 treatment (Fig. S5H). These results suggest the dependency of these alternative splicing events on PRMT5, E2F1 and MYCN.

Next, we wanted to assess the functional role of the splicing and RNA processing proteins regulated by MYCN and E2F1 (Fig. 3) in the alternatively spliced exon in *DIABLO*, *BCL2L11*, *CFLAR* and *ACIN1* RNA. For this, we manipulated the level of each protein with siRNA treatment which in CHP‐134 cells impacted on the level of exon skipping, though the effect was often seen to be gene‐specific (Fig. S6). For example, siHNRNPM or siSRSF1 treatment impacted on the alternative splicing of all four apoptotic genes in response to T1‐44 treatment (Fig. S6H,J), whilst siCPSF4 treatment impacted on the *DIABLO* alternative splicing event and did not appear to be significantly involved in the regulation of the *BCL2L11*, *ACIN1*, or *CFLAR* splicing event (Fig. S6D). These results indicate that PRMT5, E2F1 and MYCN influence the alternative RNA splicing programme of apoptotic genes through certain splicing factors and RNA processing proteins. The increased expression of the splicing and RNA processing genes, dependent on MYCN and E2F1, provides a likely explanation for the higher alternative RNA splicing activity observed in MYCN‐expressing cell lines like CHP‐134.

### 3.7 Global analysis of PRMT5 and E2F1 on alternative RNA splicing

**Fig. 6:**
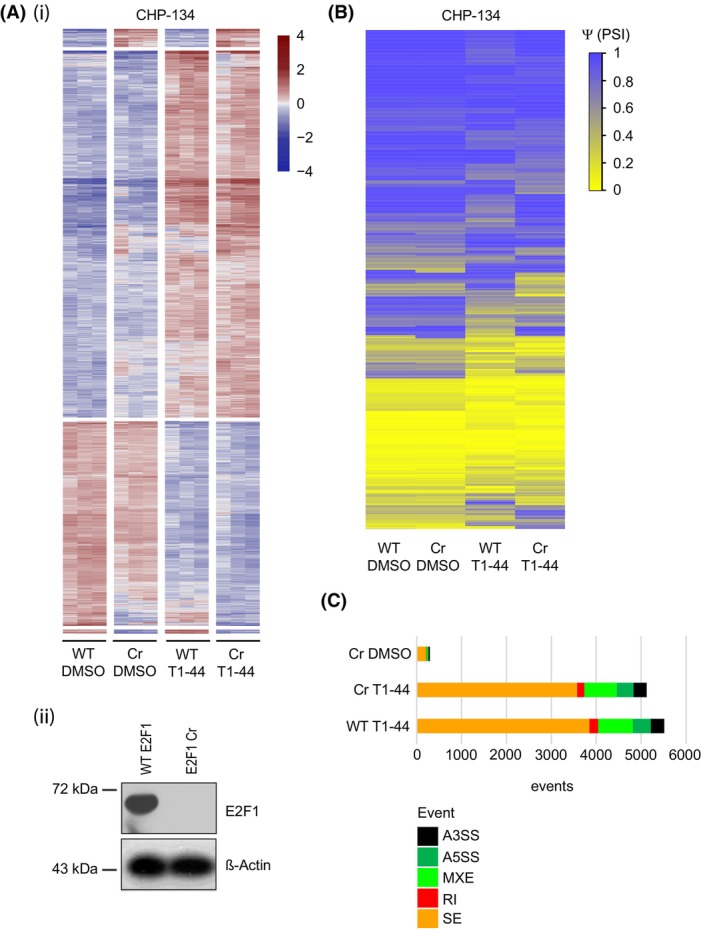
The regulation of alternative splicing events following PRMT5 inhibition by T1‐44 is E2F1 dependent. (A) (i) A heat map displaying significant differentially expressed genes (DEGs) between wild‐type (WT) E2F1 and E2F1 CRISPR (Cr) CHP‐134 cells treated with T1‐44 or control DMSO. Normalised variance stabilising transformation (vst)‐transformed gene expression values corresponding to significantly expressed genes (FDR < 0.01 and log_2_(FC) > 1.25) were mean‐centered by rows. Each row of the heatmap represents transformed expression values of one DEG across all samples (blue: low expression; red: high expression). These data were generated from three independent biological samples (*n* = 3). (ii) Immunoblot to display protein levels of E2F1 in the WT E2F1 and E2F1 Cr CHP‐134 cell lines. β‐Actin serves as a loading control for this experiment. (B) A heat map displaying values of PSI (Ψ; per cent spliced in) in wild‐type (WT) E2F1 and E2F1 CRISPR (Cr) CHP‐134 cells treated with T1‐44, corresponding to statistically significant differential splicing event changes (FDR < 0.01) with respect to the WT E2F1 CHP‐134 cell line treated with DMSO. Yellow colour represents low PSI values, and blue colour represents the high PSI values. These data were generated from three independent biological samples (*n* = 3). (C) The bar chart displays the statistically significant differential splicing events for each treatment, as compared to wild‐type (WT) E2F1 CHP‐134 cells treated with DMSO. The total number of these splicing changes corresponding to different types of splicing events is displayed in different colours. A3SS, alternative 3′ splice; A5SS, alternative 5′ splice site; MXE, mutually exclusive exons; RI, retained intron; SE, skipped/cassette exon. These data were generated from three independent biological samples (*n* = 3).

Having established that PRMT5 and E2F1 impact on gene‐specific alternative RNA splicing events, we progressed on to determine if either gene has a global influence on alternative RNA splicing events, and thus performed RNA‐seq on WT and CHP‐134 E2F1 Cr cells treated with or without T1‐44. We observed relatively minor differences in the level of differential gene expression between the WT and E2F1 Cr cells (Fig. 6A, Fig. S7A and Table S5). However, a more significant difference was apparent in alternative splicing events, particularly in cells treated with T1‐44 (Fig. 6B, Fig. S7B,C and Table S6), and there was a notable shift in both splicing events and repertoire of genes subjected to alternative splicing after T1‐44 treatment in WT compared to E2F1 Cr cells (Fig. 6C, Fig. S7B,D).

### 3.8 E2F1 influences cell sensitivity to PRMT5

**Fig. 7:**
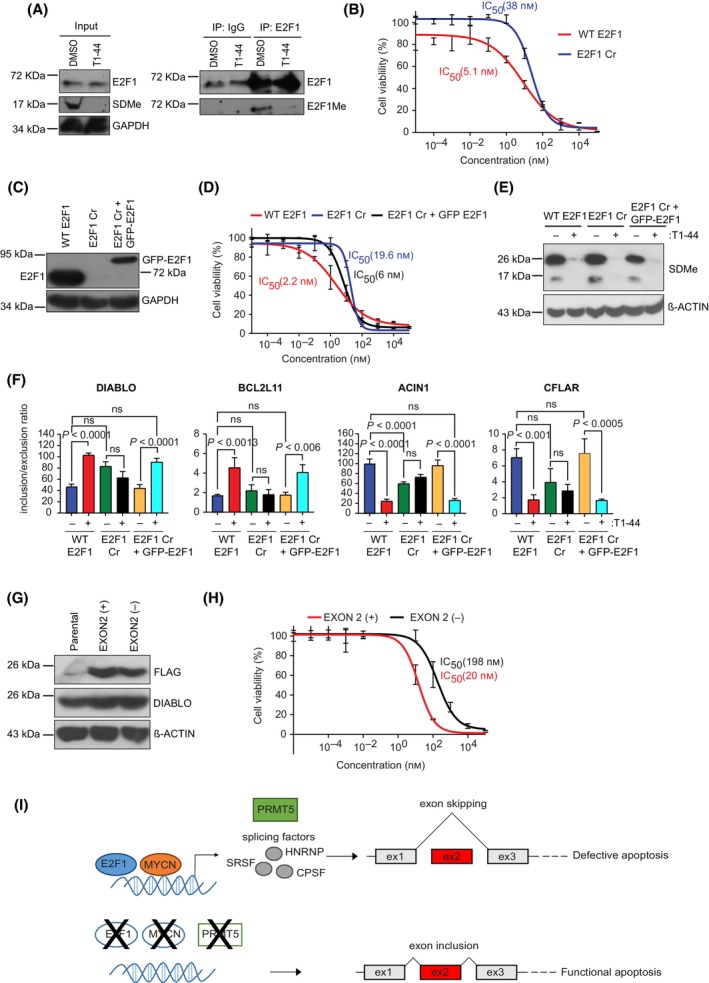
Sensitivity to PRMT5 inhibition by T1‐44 is E2F1 dependent. (A) CHP‐134 cells were treated with 200 nm T1‐44 for 48 h prior to immunoprecipitation of cell extracts with E2F1 antibodies, or non‐specific IgG. The resulting immuno‐precipitates were analysed by immunoblot using anti‐E2F1 antibodies. The blot was also probed with anti‐symmetric dimethylation (SDMe) antibodies (E2F1Me), looking specifically for a band that migrated at the same molecular weight as E2F1 (*n* = 3). (B) IC_50_ curves for wild‐type (WT) E2F1 and E2F1 CRISPR (Cr) CHP‐134 cell lines treated for 144 h with increasing T1‐44 concentrations (10^−5^–10^5^ nm), with DMSO serving as the untreated control. IC_50_ curves were determined by nonlinear regression (curve fit) using log_10_ (inhibitor) concentration versus response (three parameters) model in graphpad prism. Results represent mean percentage survival values ± SD per compound concentration; *n* = 3 independent experiments (each with three technical replicates). (C) Representative Immunoblot (*n* = 3 independent experiments) showing E2F1 expression in wild‐type (WT) E2F1 and E2F1 CRISPR (Cr) CHP‐134 cells, and in E2F1 Cr cells stably expressing ectopic wild‐type GFP‐tagged E2F1 (E2F1 Cr + GFP‐E2F1). ß‐actin served as a loading control for this experiment. (D) IC_50_ curves for the wild‐type (WT) E2F1, E2F1 CRISPR (Cr) and the E2F1 Cr with stably expressed full‐length GFP‐E2F1 cell lines treated for 144 h with increasing T1‐44 concentrations (10^−5^–10^5^ nm). DMSO treatment served as the untreated control. IC_50_ curves were determined by nonlinear regression (curve fit) using log_10_ (inhibitor) concentration versus response (three parameters) model in graphpad prism Results represent mean percentage survival values ± SD per compound concentration; *n* = 3 independent experiments (each with three technical replicates). (E) A representative immunoblot of *n* = 3 independent experiments displaying symmetric dimethylation (SDMe) protein levels in wild‐type (WT) E2F1, E2F1 CRISPR (Cr) and the E2F1 Cr cells expressing full‐length GFP‐E2F1, treated with T1‐44 (200 nm) or DMSO for 72 h. ß‐Actin served as a loading control for these experiments. (F) Changes in the differential splicing events of the apoptotic genes *DIABLO*, *BCL2L11*, *ACIN1*, and *CFLAR* upon the treatment of wild‐type (WT) E2F1, E2F1 CRISPR (Cr) and the E2F1 Cr cells stably expressing full‐length GFP‐E2F1 treated with T1‐44 or DMSO for 72 h. Results represent the mean inclusion/exclusion ratios for the skipped exons ± SD; significance was calculated with a one‐way ANOVA with Tukey's multiple comparison test; *n* = 3 independent experiments (each with three technical replicates). (G) A representative immunoblot of *n* = 3 independent experiments displaying the over‐expression of FLAG‐tagged DIABLO plasmid constructs lacking (EXON2^−^) or containing exon2 (EXON2^+^) sequence in their open reading frames (ORFs), in wild‐type CHP‐134 cells. The cells are compared against parental CHP‐134 cells that express only endogenous DIABLO. ß‐Actin served as a loading control for this experiment. (H) IC_50_ curves of ectopically expressing DIABLO (EXON 2^+^ and EXON 2^−^) CHP‐134 cell lines treated for 144 h with increasing T1‐44 concentrations (10^−5^–10^5^ nm). DMSO treatment served as the untreated controls. Curves were determined by nonlinear regression (curve fit) using log_10_ (inhibitor) concentration versus response (three parameters) model in graphpad prism Results represent mean percentage survival values ± SD per compound concentration; *n* = 3 independent experiments (each with three technical replicates). (I) Model describing the interplay between MYCN, E2F1 and PRMT5 activity, which regulate the expression of downstream target genes including those encoding for splicing factors and RNA‐binding proteins such as members of the SRSF, HNRNP, and CPSF families. These splicing factors then subsequently regulate the alternative splicing of mRNAs, including those regulated by PRMT5 activity and derived from MYCN and E2F1 target genes, for example apoptotic regulators such as DIABLO, to produce protein isoforms that are defective at driving apoptosis. In cell lines expressing low levels of MYCN, E2F1 and PRMT5, the splicing programme is altered to give rise to protein isoforms that are more efficient at driving functional apoptosis. PRMT5 inhibition also causes a consequent shift in spliced isoforms expressed from the apoptotic genes towards variants that preferentially drive apoptosis. This in part might explain the increased sensitivity to T1‐44 observed in neuroblastoma cell lines expressing high levels of MYCN, PRMT5 and E2F1.

Given that E2F1 is an important regulator of splicing events in genes required for apoptosis, we wanted to relate these observations to the effect of PRMT5 inhibition on cell death. We therefore tested whether the sensitivity of WT was any different to E2F1 Cr CHP‐134 cells upon treatment with T1‐44. Beforehand, we assessed the methylation status of E2F1 in CHP‐134 cells, which we subsequently confirmed (Fig. 7A and Fig. S7G), which is consistent with previous results [15, 16]. Treating the cells with T1‐44 resulted in the expected inhibition of the SDMe mark (Fig. 4D and Fig. S5B) and when we compared to WT cells, the E2F1 Cr cells were less sensitive to T1‐44 treatment when cell viability was measured (IC_50_ 5.1 nm compared to 38 nm; Fig. 7B, and IC_50_ 7.5 nm compared to 31 nm, Fig. S5E). Under these conditions, there were limited changes in the expression of splicing factor and RNA processing genes (Fig. S8A,B), and thus this result is consistent with a role for E2F1 in regulating cell sensitivity to apoptosis.

To confirm this role for E2F1, we assessed whether introducing ectopic E2F1 into E2F1 Cr cells would reinstate cell sensitivity to PRMT5 inhibition. Compared to the E2F1 Cr cells, stable expression of ectopic E2F1 enhanced sensitivity to T1‐44, resulting in a similar IC50 to WT cells (IC_50_ 2.2–6 nm respectively compared to 19.6 nm in the E2F1 Cr cells; Fig. 7D). In addition, the stable expression of ectopic E2F1 reinstated the exon skipping events on *DIABLO*, *BCL2L11*, *ACIN1* and *CFLAR* RNA in WT CHP‐134 cells (Fig. 7F). For example, the increased inclusion of *DIABLO* exon 2 in WT cells upon T1‐44 treatment was reinstated upon the ectopic expression of E2F1 (Fig. 7F); a similar effect was seen with *BCL2L11*, *ACIN1* and *CFLAR* RNA (Fig. 7F). These results further support the role of E2F1 in mediating the exon skipping events. To test whether the sensitivity of alternative splicing to PRMT5 activity was the result of E2F1‐mediated arginine methylation, we expressed the E2F1 derivative R113K (with an arginine to a lysine residue change at R113) [15] in E2F1 Cr cells and compared its activity to WT E2F1 (Fig. S8C–E). Compared to the expression of WT E2F1, R113K was compromised in its ability to influence alternative splicing of each gene (Fig. S8E,F); whilst the untransfected wild‐type (WT) and E2F1 Cr cells behaved as expected (Fig. S8F).

We next addressed whether the protein isoforms of DIABLO that resulted from splicing of exon 2 were of functional importance. DIABLO is a mitochondria‐associated protein which binds to inhibitor of apoptosis proteins (IAPs), thus freeing caspases to activate apoptosis [50]. We therefore prepared stable CHP‐134 cells expressing the isoforms of DIABLO with or without an intact exon 2 (Fig. 7G) and measured the effect on cell viability and apoptosis. The DIABLO isoform with an intact exon 2 was more effective at conferring sensitivity to T1‐44 treatment when cell viability or sub‐G1 apoptotic cells were measured (Fig. 7H and Fig. S8G), suggesting that the exon2 skipping event impacts on the biological activity of DIABLO. In addition, when we reduced the level of endogenous DIABLO with gene‐specific siRNA (Fig. S8H), we observed a significant decrease in the level of apoptosis that occurred following treatment with T1‐44 (Fig. S8I). These results suggest a role for DIABLO and its alternative RNA splicing in mediating apoptosis in response to PRMT5 inhibition.

### 3.9 Relevance to clinical disease

It was of interest to relate the expression of the splicing and RNA processing genes to *PRMT5*, *E2F1* and *MYCN* in clinical disease. For this, we interrogated the human neuroblastoma expression data in SEQC (derived from *n* = 498 biopsies), Westermann (*n* = 597) and the neuroblastoma PPTX dataset that contains expression data on neuroblastoma PDX models (*n* = 33) [24, 41, 42]. We observed a moderate to strong correlation between the expression of *PRMT5*, *E2F1* and *MYCN* and the splicing factor genes *CPSF3*, and *4*, *HNRNPA3*, *HNRNPM* and *SRSF1* and *3* (Fig. S9A–C). It was of further interest that we detected the exon2 RNA splice variants derived from *DIABLO*, *BCL2L11*, *CFLAR* and *ACIN1* in the TARGET neuroblastoma data set [5], although expression data for relevant spliced transcripts was only available for *ACIN1* and *CFLAR* (Fig. S9D,E and Table S7). Here, the *CFLAR* RNA variant containing exon2 (namely, exon inclusion) was seen to positively correlate with high levels of the splicing factor *SRSF1*, as well as with *MYCN* (Fig. S9D). A similar situation was observed for the *ACIN1* RNA variant lacking exon2 (exon excluded form) where increased expression of the splicing factor genes coincided with decreased levels of the exon2 excluded form (Fig. S9E), as predicted from the higher exon inclusion ratio observed in control siRNA treated cells (Fig. S6H,J,L). These results derived from analysing expression levels in clinical biopsies are therefore compatible with similar RNA splicing variants occurring in clinical disease.

## 4 Discussion

Neuroblastoma is a highly problematic paediatric cancer with limited treatment options. Other than *MYCN*, which is often over‐expressed in poor prognosis disease [5], there is relatively little information on any cancer‐relevant pathways that distinguish between each prognostic subgroup. With this background in mind, we have sought to understand the biological and molecular role of PRMT5 and E2F1 in NB, and most importantly the interplay with MYCN. This question was of interest because previous studies have established that PRMT5 drives multiple oncogenic processes and is over‐expressed in diverse types of tumours [17] including neuroblastoma [17]. On the other hand, although E2F1 was initially described as a transcription factor which controls cell cycle‐dependent gene expression, more recent studies have defined a much wider role in both transcriptional control and alternative RNA splicing [12, 13, 19]. Most importantly, E2F1 is a significant and specific methylation target for PRMT5, a methylation event which alters the biochemical and biological properties of E2F1 [13, 14, 15, 16]. Since E2F1 is a biological target for PRMT5, and because *PRMT5* is over‐expressed in neuroblastoma, we reasoned that an important relationship may exist between the two genes that contributes to the aetiology of neuroblastoma.

A significant finding from the current study, which arose through measuring gene expression in clinically annotated biobanks of human NB biopsies, has established for the first time that *MYCN*, *PRMT5* and *E2F1* are frequently co‐expressed at high levels in poor prognosis high‐risk disease. The expression of a number of other genes has been linked to the different prognostic groups in NB [4]. However, we believe that the co‐expression of *MYCN*, *PRMT5* and *E2F1* is of importance because the over‐expression is underpinned by a functional relationship between each protein product, as discussed below. The over‐expression of *MYCN*, *PRMT5* and *E2F1* not only provides a potential poor prognosis signature but equally suggests a rationale for treating this group of patients with drugs that target the pathway.

A genome‐wide analysis in high and low‐expressing cell lines identified quite significant expression differences in the role of PRMT5 on both transcription and RNA splicing activity. Using RNA‐seq to assess genome‐wide expression, we found differences in the expression profile in CHP‐134 cells compared to GI‐ME‐N cells, where transcription in high‐expressing CHP‐134 cells was widely affected by PRMT5 activity compared to GI‐ME‐N cells, arguing that the high‐level expression of PRMT5 reflected its significant influence on gene expression. We also noticed that PRMT5 impacted on alternative RNA splicing, which again was far more marked in CHP‐134 cells compared to GI‐ME‐N cells. This observation is compatible with previous reports suggesting that PRMT5 and E2F1 target and influence the alternative RNA splicing programme in tumour cells [13].

Mining the expression data led us to an interesting speculation, namely that pathways impacted by PRMT5 in NB cells were connected with apoptosis. This prompted us to dive deeper into the data sets in an attempt to identify candidate genes that could theoretically be targets for PRMT5 activity. We noted that apoptosis is frequently defective in tumour cells [52] and thus we reasoned that genes involved in apoptosis could be rendered less active in NB cells, possibly through a mechanism that involved aberrant alternative splicing. Consequently, we identified a number of genes required for apoptosis, namely *DIABLO*, *BCL2L11* (*BIM*), *ACIN1* and *CFLAR*, each encoding protein components that contribute to efficient apoptotic programme [42, 44, 49]. The RNAs derived from these genes were of interest because they had either an exon skipped or included that would be likely to lead to functional impairment of protein function, since the exons encoded protein domains of potential functional importance [44, 48, 49]. This idea was confirmed for *DIABLO*, where an analysis of the variant protein isoforms arising from the alternatively spliced exon 2, was shown to influence its apoptotic activity. The alternatively spliced exon 2 affected its mitochondrial targeting signal, located in the N‐terminal region [50] potentially impacting on the mitochondrial location of DIABLO and therefore its apoptotic activity. The exon‐skipping event observed in *DIABLO* and the other genes was not only dependent on PRMT5, but also MYCN and E2F1. It is possible therefore that the negative impact on apoptosis that correlates with high MYCN, PRMT5 and E2F1 contributes to the high‐risk poor prognosis disease profile which also correlates with the over‐expression of these genes.

Defects in gene‐specific alternative splicing events are well established to occur in human tumours, where the activity of genes connected with cell growth and death has been shown to be severely impacted by alternative splicing [53]. For example, BCL2L1 generates two protein isoforms in consequence to alternative splicing, BCL‐xL and BCL‐xS, which have opposing functions in apoptosis; the first prevents apoptosis while the latter promotes it [54]. Further, the control of caspase2 reflects an alternative splicing event which produces two protein isoforms, either with pro‐apoptotic or anti‐apoptotic activity [55].

In addition, our results suggest a plausible mechanism through which the heightened level of alternative splicing occurs. Thus, MYCN and E2F1 are both transcription factors that we detected in the promoter region of a variety of RNA splicing factor and RNA processing genes, and MYCN activity was required for their expression. PRMT5 has been shown to methylate diverse substrate proteins involved in RNA splicing [56] and therefore we suggest that the increased level of splicing factors provides substrates for PRMT5 which leads to aberrant splicing events in relevant genes (like *DIABLO*) that subsequently act to promote the malignant phenotype (Fig. 7I). Although we cannot rule out a role for PRMT5 in other processes, our results do minimally implicate alternative splicing as an anti‐apoptotic mechanism in NB, and suggest a role for MYCN, PRMT5 and E2F1 in facilitating this process. Mechanistically, because PRMT5 fostered exon skipping in key apoptotic genes, leading to reduced sensitivity to apoptosis, our results further imply that the heightened level of alternative splicing has functional relevance in cell survival and potentially contributes to the high‐risk poor prognosis disease profile. In tumours where *MYCN*, *PRMT5* and *E2F1* are expressed at lower levels and splicing activity is similarly reduced, we suggest that other PRMT5 independent mechanisms contribute to the malignant phenotype. In this respect, it is noteworthy that in clinical neuroblastoma the expression of splicing and RNA processing factor genes was seen to coincide with *PRMT5*, *E2F1* and *MYCN* levels, and similarly with some of the exon skipping events in the apoptotic genes.

Our mechanistic model combines the observations made here and potentially explains the interplay between each protein. We developed our model based on results derived from NB cell lines which harboured a similar high and low expression profiles of MYCN, PRMT5 and E2F1. Thus, a significant caveat must bear in mind the experimental systems used to generate the data, and the potential limitations to clinical disease that *in vitro* studies on cell lines necessarily suffer from. Testing the predictions from the results described here in animal models that recapitulate the pathology of neuroblastoma will be an important next step. Nevertheless, our results do suggest a provocative model that integrates the activity of MYCN, PRMT5 and E2F1 which can be tested in further studies.

## 5 Conclusion

In conclusion, our results shed light on a new molecular mechanism that could contribute to high‐risk NB disease and provide mechanistic insights into how this is achieved. Most importantly, our studies highlight PRMT5 as a potential therapeutic target in high‐risk disease, because inhibition of PRMT5 reinstated a sensitive apoptotic programme which led to cell death. Specifically, we suggest that MYCN and E2F1 increase the levels of splicing factors which in turn are targeted by PRMT5 to reprogramme alternative RNA splicing in a fashion that contributes to malignant disease, achieved in part through decreased activity of apoptotic pathways. Our results in turn suggest PRMT5‐based inhibition as a viable therapeutic strategy for treating high‐risk disease.

## Conflict of interest

The authors declare no conflict of interest.

## Author contributions

Conceptualisation – NBLT; Data curation – LTB‐E, ASa, AK; Formal analysis – LTB‐E, ASa, AK, SMC, GA, ASh; Funding acquisition – NBLT; Investigation – LTB‐E, GA; Methodology – LTB‐E, ASa, AK, SMC; Project administration – NBLT; Software – ASa, AK; Supervision – NBLT; Validation – LTB‐E, SMC, GA; Writing—original draft – NBLT; Writing—review & editing – NBLT, LTB‐E, SMC.

### Peer review

The peer review history for this article is available at https://www.webofscience.com/api/gateway/wos/peer‐review/10.1002/1878‐0261.13702.

## Supporting information


**Fig. S1.** High expression of *PRMT5*, *E2F1* and *MYCN* correlates with poor prognosis in neuroblastoma.
**Fig. S2.** Gene ontology analysis of differentially expressed and differentially spliced genes.
**Fig. S3.** Splicing factors are transcriptional targets for E2F1 and MYCN in neuroblastoma cell lines.
**Fig. S4.** Splicing factors and apoptotic genes are transcriptional targets for E2F1 and MYCN in neuroblastoma cell lines.
**Fig. S5.** Changes in the differential splicing events of the apoptotic genes, *DIABLO*, *BCL2L11*, *ACIN1*, and *CFLAR* upon treatment with T1‐44 in neuroblastoma cell lines.
**Fig. S6.** Specific splicing factors regulate splice events in apoptotic target genes.
**Fig. S7.** Analysis of differentially spliced genes in E2F1 CRISPR CHP‐134 cells treated with PRMT5 inhibitor T1‐44.
**Fig. S8.** Analysis of differential splicing events in E2F1 CRISPR CHP‐134 cell lines over‐expressing wild‐type or methylation‐defective E2F1.
**Fig. S9.** Correlation in expression between *MYCN*, *E2F1*, *PRMT5* and splicing factors in human neuroblastoma samples.

**Table S1.** Details of all primer sequences used in this study.

**Table S2.** A list of neuroblastoma cell lines used in this study, and a summary of their corresponding *MYCN*, *PRMT5* and *E2F1* mRNA expression levels and IC_50_ values in response to T1‐44 and GSK581 treatment.

**Table S3.** DEG analysis on CHP‐134 and GI‐ME‐N cells treated with T1‐44 (compared to DMSO).

**Table S4.** rMATS differential splicing analysis on CHP‐134 and GI‐ME‐N cells treated with T1‐44 (compared to DMSO).

**Table S5.** DEG analysis of CHP‐134 WT E2F1 and E2F1 Cr cells treated with T1‐44 (compared to WT E2F1 cells treated with DMSO).

**Table S6.** rMATS differential splicing analysis of CHP‐134 WT E2F1 and E2F1 Cr cells treated with T1‐44 (compared to WT E2F1 cells treated with DMSO).

**Table S7.** TARGET neuroblastoma sample expression data.

## Data Availability

Four sets of microarray data from the neuroblastoma patient cohorts were downloaded from the open access R2 database (R2: Genomics analysis and visualisation platform [http://r2.amc.nl]). Publicly available data from the GEO databases (GSE45547, GSE73517, GSE3960 and GSE62564 RNA‐seq data series were used). RNA‐seq datasets and gene expression data generated in this manuscript have been deposited in the National Center for Biotechnology Information's (NCBI) Gene expression Omnibus (GEO) and are accessible through GEO Series accession number GSE243989. All additional data is included in the Supporting Information.
