# Supplementary material for: Sustained cancer‐relevant alternative RNA splicing events driven by PRMT5 in high‐risk neuroblastoma
Source: Mol Oncol. 2024 Jul 17;19(3):741–63. doi: 10.1002/1878-0261.13702 (PMC11887678; doi:10.1002/1878-0261.13702)
Supplement: Supplementary file 1 — Fig. S1. High expression of PRMT5, E2F1 and MYCN correlates with poor prognosis in neuroblastoma. Fig. S2. Gene ontology analysis of differentially expressed and differentially spliced genes. Fig. S3. Splicing factors are transcriptional targets for E2F1 and MYCN in neuroblastoma cell lines. Fig. S4. Splicing factors and apoptotic genes are transcriptional targets for E2F1 and MYCN in neuroblastoma cell lines. Fig. S5. Changes in the differential splicing events of the apoptotic genes, DIABLO, BCL2L11, ACIN1, and CFLAR upon treatment with T1‐44 in neuroblastoma cell lines. Fig. S6. Specific splicing factors regulate splice events in apoptotic target genes. Fig. S7. Analysis of differentially spliced genes in E2F1 CRISPR CHP‐134 cells treated with PRMT5 inhibitor T1‐44. Fig. S8. Analysis of differential splicing events in E2F1 CRISPR CHP‐134 cell lines over‐expressing wild‐type or methylation‐defective E2F1. Fig. S9. Correlation in expression between MYCN, E2F1, PRMT5 and splicing factors in human neuroblastoma samples. [file MOL2-19-741-s002.pdf]

Supplementary Figure 1

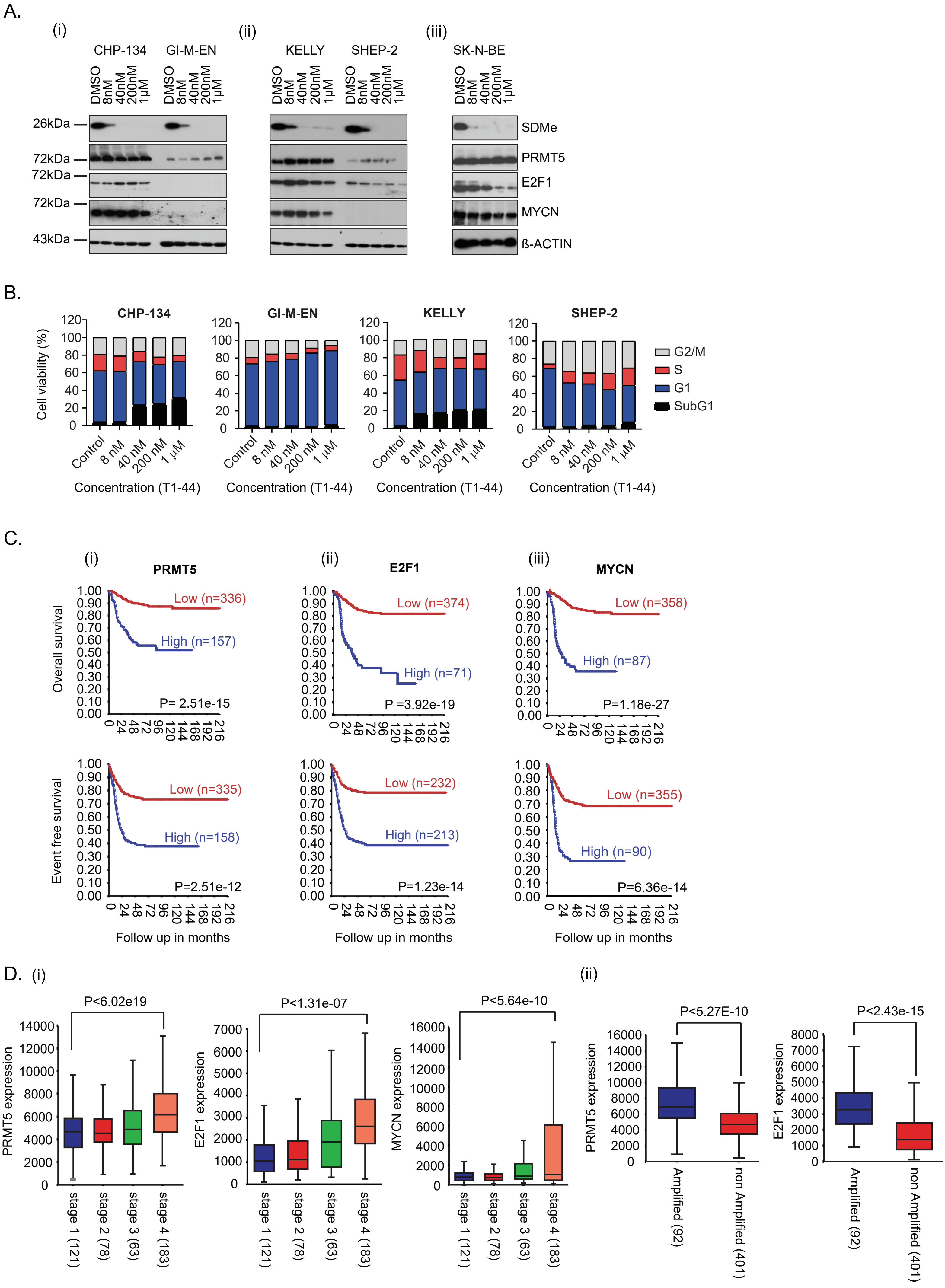

SI Figure 1: High expression of *PRMT5*, *E2F1* and *MYCN* correlates with poor prognosis in neuroblastoma.

A (i-iii) Immunoblots to display protein levels of symmetric dimethylation (SDMe), PRMT5, E2F1 and MYCN levels in three sensitive cell lines CHP-134, KELLY and SK-N-BE and two insensitive cell lines GI-ME-N, SHEP-2 and treated for 144 hours with four increasing T1-44 concentrations (8nM, 40nM, 200nM and 1µM) with DMSO serving as a control. SDMe served as a control for PRMT5 activity.

B Total cell cycle analysis depicting the Sub-G1, G1, S and G2/M phase of the cell cycle of CHP-134, GI-ME-N, KELLY, and SHEP-2 cells stained with propidium iodide and treated with increasing concentrations of T1-44 for 144h (8nM, 40 nM, 200nM and 1µM) with DMSO serving as a control for the treated cell lines.

C Overall (above) and event-free (below) patient survival probability in stage 1-stage 4 neuroblastoma patients in the SEQC databases (n=498 patients) with respect to the *PRMT5*, *E2F1* and *MYCN* mRNA expression in these tumours. *P* values were calculated with a log-rank test for survival curves.

D (i) Correlation of *PRMT5* (P<6.02e-19), *E2F1* (P<1.3-07) and *MYCN* (P<5.64e-10) expression levels between INSS (International Neuroblastoma Staging System) neuroblastoma stages with stage 1 (n=121), stage 2 (n=78), stage 3 (n=63) and stage 4 (n=183). Kruskal–Wallis with Dunn’s multiple comparisons test was used to determine *p* values, which were corrected with the Bonferonni correction method. (ii) Box plot correlational analysis of *PRMT5* (P<5.27e-10) and *E2F1* (P<2.43e-15) mRNA in the *MYCN* amplified (n=550) and non-*MYCN* amplified (n=93) neuroblastoma tumours. *P* values were calculated with a two-sided Wilcoxon rank-sum test for boxplots. Boxplot centre represents mean, the box represents SD, and whiskers represent minimum and maximum.

Supplementary Figure 2

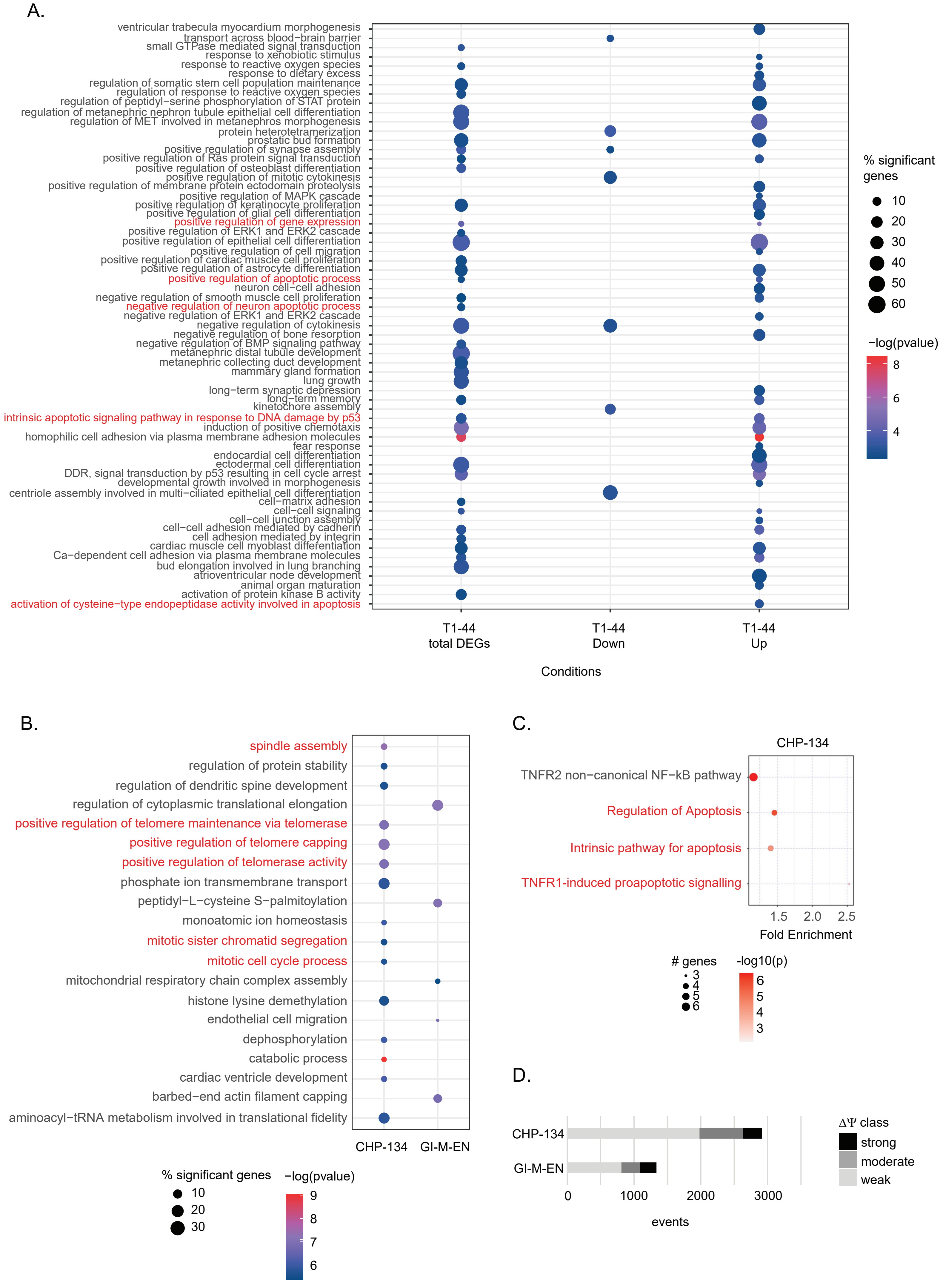

SI Figure 2: Gene ontology analysis of differentially expressed and differentially spliced genes

A The topGO package was used to identify gene ontology biological process (GO:BP) terms enriched for genes that are differentially expressed in CHP-134 cells treated with T1-44 (log_2(FC) > 1, padj < 0.01). The analysis was performed on the total differentially expressed gene (DEG) list, and on up-regulated and down-regulated gene lists separately. Terms related to gene expression and apoptotic processes are highlighted in red.

B The topGO package was used to uncover gene ontology biological process (GO:BP) terms enriched for genes that are alternatively spliced in CHP-134 and GI-ME-N cells treated with T1-44. Terms related to telomere maintenance and the mitotic cell cycle are highlighted in red.

C Functional characterisation of CHP-134 differentially spliced genes using pathfindR. Terms related to apoptotic processes are highlighted in red.

D Differential changes in splicing between T1-44 treated and DMSO control treated CHP-134 and GI-ME-N cell lines. The bar chart displays the total number of splicing events that score as strong ($\Delta\Psi$ > 0.5), moderate ($\Delta\Psi$ between 0.3-0.5), or weak ($\Delta\Psi$ between 0.1-0.3) splice events. Events with a $\Delta\Psi$ < 0.1 were excluded from the analysis.

Supplementary Figure 3

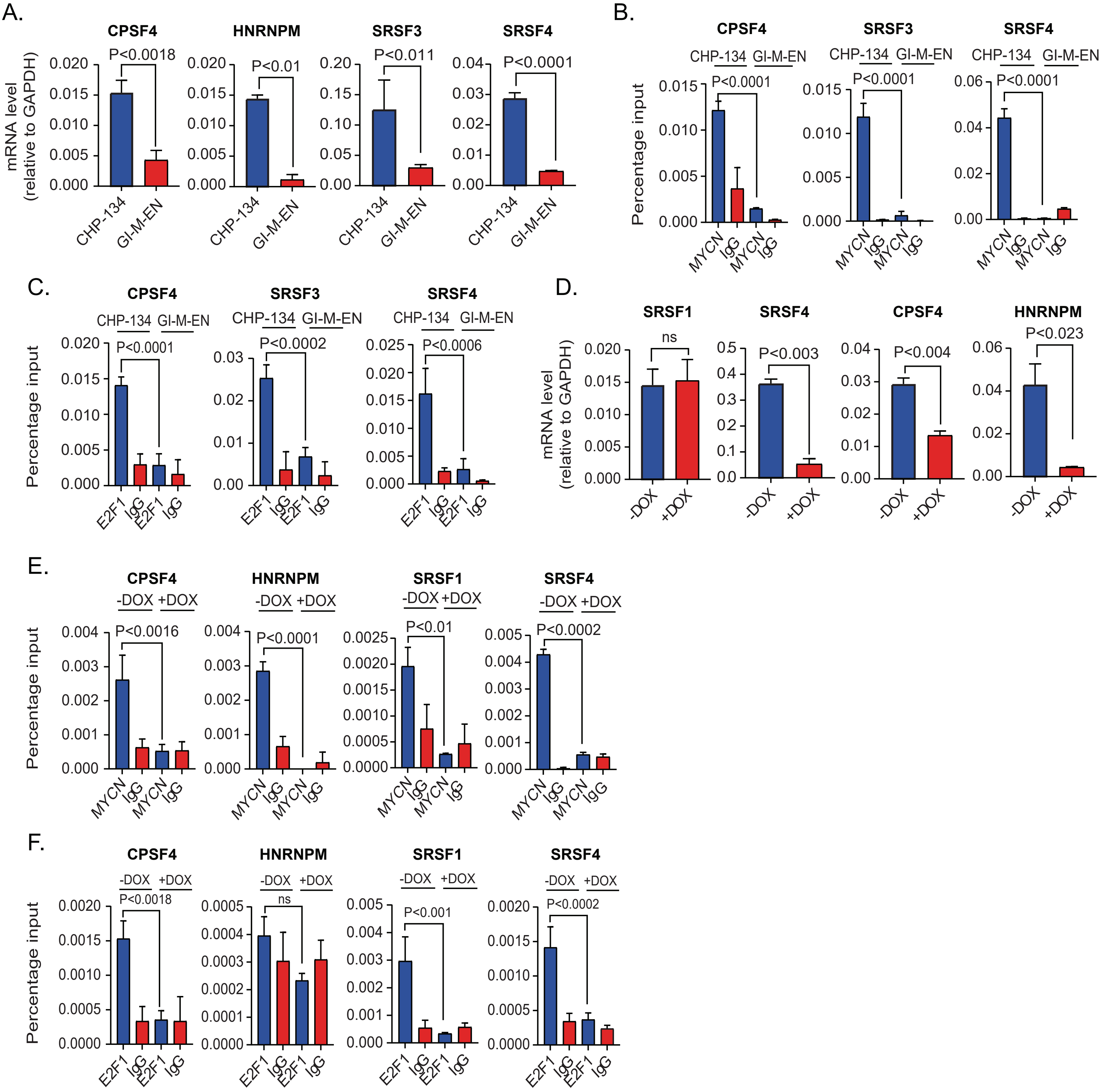

SI Figure 3: Splicing factors are transcriptional targets for E2F1 and MYCN in neuroblastoma cell lines

A mRNA expression of splicing factors *CPSF4*, *SRSF3-4* and *HNRNPM* in CHP-134 and GI-ME-N cell lines. Results represent mean expression values ±SD; n=3 independent experiments (each with three technical replicates); significance was calculated with an unpaired *t*-test.

B- C Chromatin immunoprecipitation **(**ChIP) assays denoting the binding affinity of MYCN and E2F1 transcription factors on the promoter region of the splicing factor genes *CPSF4* and *SRSF3-4* in CHP-134 and GI-ME-N cell lines. Results represent mean percentage enrichment values ±SD; significance was calculated with a one-way ANOVA with Tukey's multiple comparison test; n=3 independent experiments (each with three technical replicates).

D mRNA expression of splicing factors *SRSF1*, *4*, *CPSF4* and *HNRNPM* in in the SHEP-21N Tet-off cell line overexpressing MYCN (-DOX) and in the SHEP-21N cell line with decreased MYCN expression (+DOX for 72h). Results represent mean expression values ±SD; n=3 independent experiments (each with three technical replicates); significance was calculated with an unpaired *t*-test.

E-F Chromatin immunoprecipitation (ChIP) assays denoting the binding affinity of the MYCN and E2F1 transcription factors on the promoter region of splicing factors *CPSF4*, *SRSF1*, *4* and *HNRNPM* in the SHEP-21N Tet-off cell line overexpressing MYCN (-DOX) and in the SHEP-21N cell line with decreased MYCN expression (+DOX for 72h). Results represent the mean percentage enrichment values ±SD; significance was calculated with a one-way ANOVA with Tukey's multiple comparison test; n=3 independent experiments (each with three technical replicates).

Supplementary Figure 4

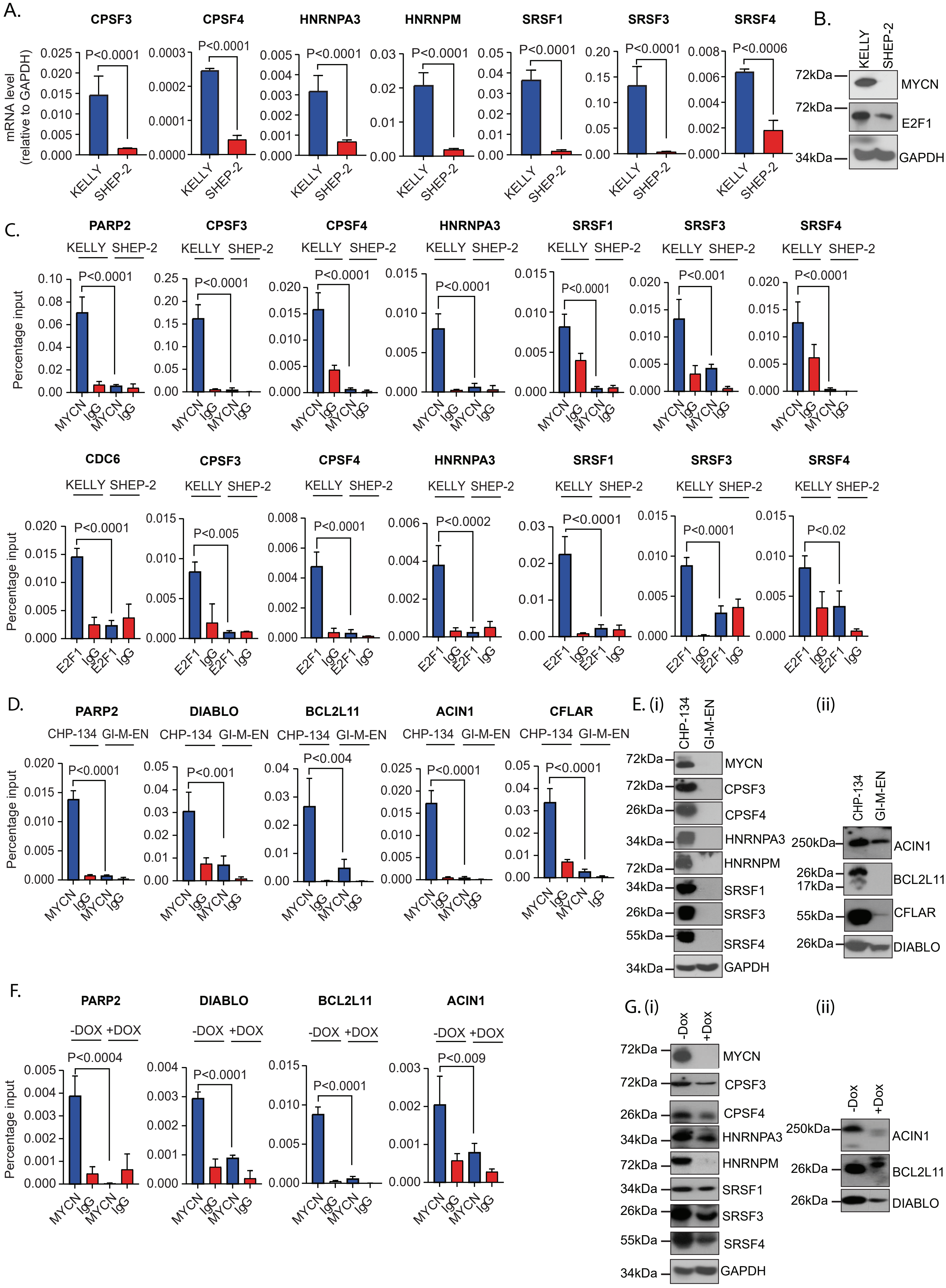

SI Figure 4: Splicing factors and apoptotic genes are transcriptional targets for E2F1 and MYCN in neuroblastoma cell lines

A mRNA expression of seven splicing factors *CPSF3-4*, *SRSF1*, *3*, *4*, *HNRNPA3* and *HNRNPM* in KELLY and SHEP-2 cell lines. Results represent mean expression values ±SD; n=3 independent experiments (each with three technical replicates); significance was calculated with an unpaired *t*-test.

B Immunoblot displaying the protein expression levels of MYCN and E2F1 in the KELLY and SHEP-2 cell lines. GAPDH served as a loading control for this experiment.

C Chromatin immunoprecipitation (ChIP) assays denoting the binding affinity of MYCN and E2F1 transcription factors on the promoter region of the splicing factor genes *CPSF3-4*, *SRSF1*, *3*, *4* and *HNRNPA3* in KELLY and SHEP-2 cell lines. *CDC6* and *PARP2* served as positive control for the binding affinity of E2F1 and MYCN respectively. Results represent mean percentage enrichment values ±SD; significance was calculated with a one-way ANOVA with Tukey's multiple comparison test; n=3 independent experiments (each with three technical replicates).

D Chromatin immunoprecipitation (ChIP) assays denoting the binding affinity of the MYCN transcription factor on the promoter region of apoptotic genes *DIABLO*, *BCL2L11*, *CFLAR* and *ACIN1* for the CHP-134 and the GI-ME-N cell lines. *PARP2* served as a positive control for the binding of MYCN. Results represent the mean percentage enrichment values ±SD; significance was calculated with a one-way ANOVA with Tukey's multiple comparison test; n=3 independent experiments (each with three technical replicates).

E (i) Immunoblot displaying protein expression levels of seven splicing factors CPSF3-4, SRSF1, 3, 4, HNRNPA3 and HNRNPM and (ii) the apoptotic genes ACIN1, BCL2L11, CFLAR and DIABLO between CHP-134 and GI-ME-N cell lines. GAPDH served as a loading control for this experiment.

F Chromatin immunoprecipitation (ChIP) assays denoting the binding affinity of the MYCN transcription factor on the promoter region of apoptotic genes *DIABLO*, *BCL2L11* and *ACIN1* in the SHEP-21N Tet-off cell line overexpressing MYCN (-DOX) and in the SHEP-21N cell line with decreased MYCN expression (+DOX for 72h). *PARP2* served as a positive control for the binding of MYCN. Results represent the mean percentage enrichment values ±SD; significance was calculated with a one-way ANOVA with Tukey's multiple comparison test; n=3 independent experiments (each with three technical replicates).

G (i) Immunoblot displaying protein expression levels of seven splicing factors CPSF3-4, SRSF1, 3, 4, HNRNPA3 and HNRNPM and (ii) the apoptotic genes ACIN1, BCL2L11 and DIABLO between the SHEP21N cell line overexpressing MYCN (-DOX) and the SHEP21N cell line with decreased MYCN expression (+DOX for 72h). GAPDH served as a loading control for this experiment.

Supplementary Figure 5

A.

Coordinates	pValue	psi_44	psi_DMSO	dpsi	Gene	Event
chr12_122710511_122711200_122709058_122709191_122711902_122712068	1.06E-04	0.734333333	0.942	-0.208	DIABLO	SE
chr2_111911356_111911481_111881322_111881716_111921709_111921808	8.45E-04	0.766	0.328666667	0.437	BCL2L11	SE
chr14_23540333_23540395_23538684_23538826_23540635_23540747	0	0.212666667	0.466333333	-0.254	ACIN1	SE
chr2_201994451_201994869_201980826_201981204_201997751_201997825	5.40E-06	0.661666667	1	-0.338	CFLAR	SE

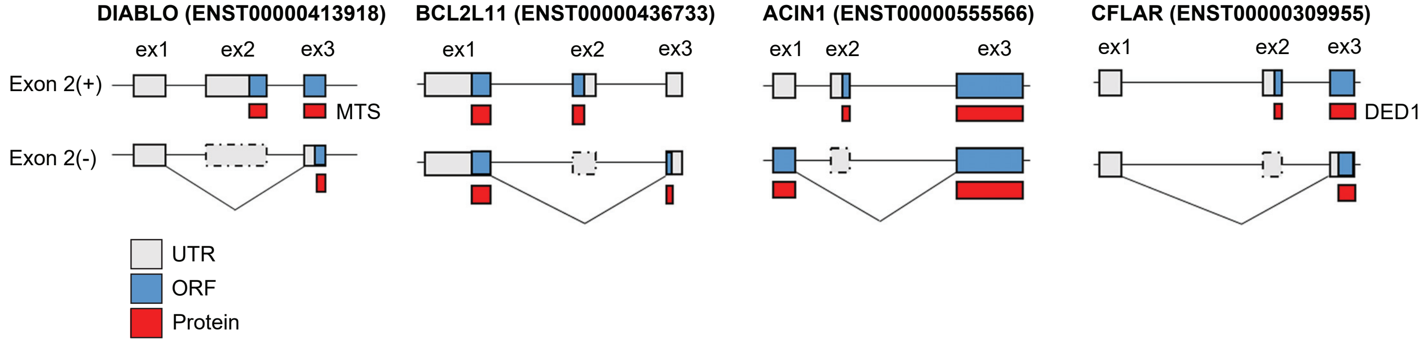

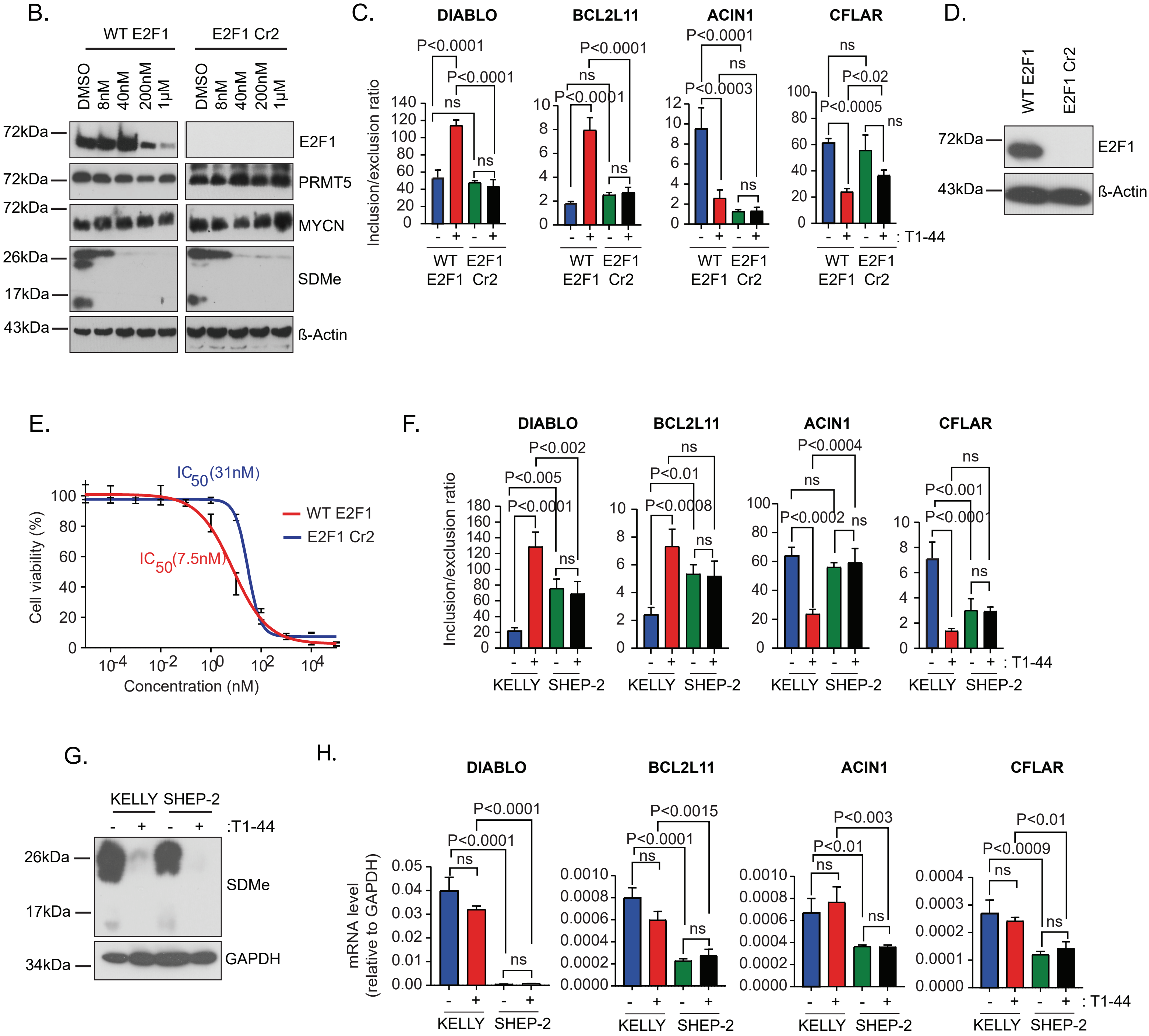

SI Figure 5: Changes in the differential splicing events of the apoptotic genes*, DIABLO, BCL2L11, ACIN1*, and *CFLAR* upon treatment with T1-44 in neuroblastoma cell lines

A The table displays part of the output from the replicative multivariate analysis of transcript splicing (rMATS) analysis performed on CHP-134 cells treated with T1-44 or DMSO. Coordinates for each of the splicing events identified in *DIABLO*, *BCL2L11*, *ACIN1* and *CFLAR* are displayed, along with percent spliced in (PSI) values in each treatment and the delta psi. Below the table is a schematic representation of the differential splice events in each gene. Exon structure for the skipped exon and flanking exons is displayed, with untranslated regions (UTR) and the open reading frame (ORF) marked in grey and blue respectively. The predicted impact of each splicing event on the ORF and derived protein sequence (displayed in red) are also included. If the skipped exon is known to encode for an amino acid sequence contributing to an annotated protein domain, this is also indicated. MTS: mitochondrial targeting signal; DED1: Death effector domain 1.

B Immunoblot displaying successful E2F1 knockout in a second E2F1 CRISPR clone (Cr2), as compared to wild-type (WT) E2F1 CHP-134 cells. Cells were treated with increasing concentrations of T1-44 and symmetric dimethylation (SDMe) was used as a marker for inhibition of PRMT5 activity. ß-actin served as a loading control for this experiment.

C Changes in the differential splicing events of the apoptotic genes *DIABLO*, *BCL2L11*, *ACIN1*, and *CFLAR* upon treatment of wild-type (WT) E2F1 and the E2F1 CRISPR clone 2 (Cr2) cell lines with T1-44 for 72 hrs. Results represent the mean inclusion/exclusion ratios for the skipped exon events ±SD; significance was calculated with a one-way ANOVA with Tukey's multiple comparison test; n=3 independent experiments (each with three technical replicates).

D Immunoblot displaying E2F1 and β-actin expression levels in wild-type (WT) E2F1 and E2F1 CRISPR (Cr2) CHP134 cells.

E IC_{50} curves of the wild-type (WT) E2F1 and the E2F1 CRISPR clone 2 (cr2) cell lines treated for 144h with increasing T1-44 concentrations (10^{-5} nM-10^{5} nM) and DMSO serving as the untreated control. Curves were determined by nonlinear regression (curve fit) using log_{10} (inhibitor) concentration versus response (three parameters) model in GraphPad Prism.

F Changes in the differential splicing events of the apoptotic genes *DIABLO*, *BCL2L11*, *ACIN1*, and *CFLAR* upon the treatment of KELLY and SHEP-2 cell lines with T1-44 for 72 hrs. Results represent the mean inclusion/exclusion ratios for the skipped exon events ±SD; significance

was calculated with a one-way ANOVA with Tukey's multiple comparison test; n=3 independent experiments (each with three technical replicates).

G Immunoblot displaying symmetric dimethylation (SDMe) levels in KELLY and SHEP-2 cell lines treated with and without T1-44 (200nM) for 72h, as a marker for inhibition of PRMT5 activity. GAPDH served as a loading control for these experiments.

H mRNA expression levels relative to *GAPDH* for the apoptotic genes *DIABLO*, *BCL2L11*, *ACIN1* and *CFLAR* in KELLY and SHEP-2 cells treated with and without T1-44 (200nM) for72h. Results represent the mean expression values ±SD; significance was calculated with a one-way ANOVA with Tukey's multiple comparison test; n=3 independent experiments (each with three technical replicates).

Supplementary Figure 6.

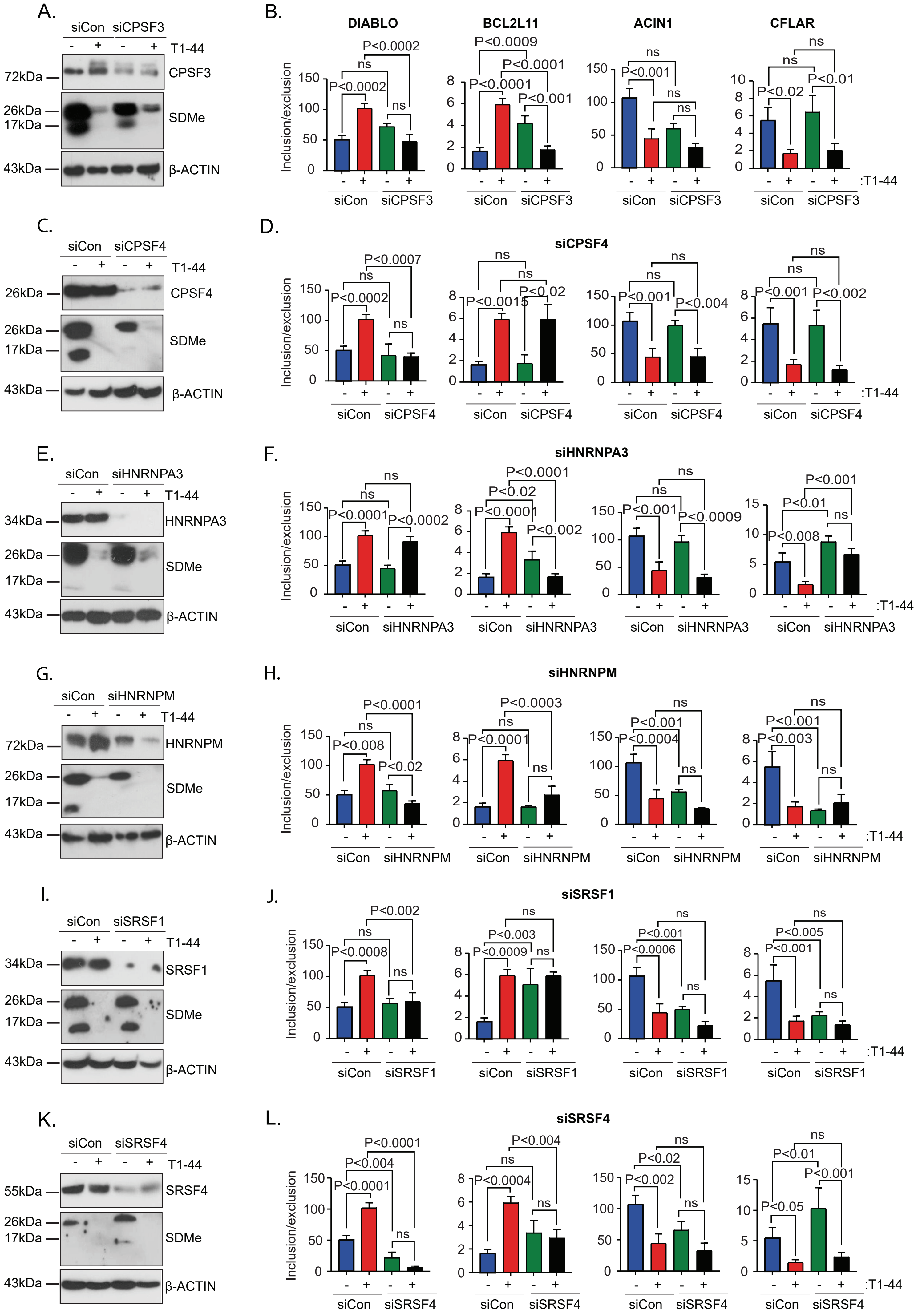

SI Figure 6: Specific splicing factors regulate splice events in apoptotic target genes

A Immunoblot showing siRNA knockdown of the CPSF3 splicing factor in CHP-134 cells treated with T1-44 (200nM) or DMSO for 72h. Symmetric dimethylation (SDMe) served as a marker for PRMT5 activity and ß-Actin was used as a loading control for this experiment.

B Changes in the differential splicing events of apoptotic genes *DIABLO*, *BCL2L11*, *ACIN1*, and *CFLAR* in CHP-134 cells treated with siCPSF3 or siControl and T1-44 or DMSO for 72 h. Results display the mean inclusion/exclusion ratios for each of the skipped exons ±SD; significance was calculated with a one-way ANOVA with Tukey's multiple comparison test; n=3 independent experiments (each with three technical replicates).

C Immunoblot showing siRNA knockdown of the CPSF4 splicing factor in CHP-134 cells treated with T1-44 (200nM) or DMSO for 72h. Symmetric dimethylation (SDMe) served as a marker for PRMT5 activity and ß-Actin was used as a loading control for this experiment.

D Changes in the differential splicing events of apoptotic genes *DIABLO*, *BCL2L11*, *ACIN1*, and *CFLAR* in CHP-134 cells treated with siCPSF4 or siControl and T1-44 or DMSO for 72 h. Results display the mean inclusion/exclusion ratios for each of the skipped exons ±SD; significance was calculated with a one-way ANOVA with Tukey's multiple comparison test; n=3 independent experiments (each with three technical replicates).

E Immunoblot showing siRNA knockdown of the HNRNPA3 splicing factor in CHP-134 cells treated with T1-44 (200nM) or DMSO for 72h. Symmetric dimethylation (SDMe) served as a marker for PRMT5 activity and ß-Actin was used as a loading control for this experiment.

F Changes in the differential splicing events of apoptotic genes *DIABLO*, *BCL2L11*, *ACIN1*, and *CFLAR* in CHP-134 cells treated with siHNRNPA3 or siControl and T1-44 or DMSO for 72 h. Results display the mean inclusion/exclusion ratios for each of the skipped exons ±SD; significance was calculated with a one-way ANOVA with Tukey's multiple comparison test; n=3 independent experiments (each with three technical replicates).

G Immunoblot showing siRNA knockdown of the HNRNPM splicing factor in CHP-134 cells treated with T1-44 (200nM) or DMSO for 72h. Symmetric dimethylation (SDMe) served as a marker for PRMT5 activity and ß-Actin was used as a loading control for this experiment.

H Changes in the differential splicing events of apoptotic genes *DIABLO*, *BCL2L11*, *ACIN1*, and *CFLAR* in CHP-134 cells treated with siHNRNPM or siControl and T1-44 or DMSO for 72 h. Results display the mean inclusion/exclusion ratios for each of the skipped exons ±SD;

significance was calculated with a one-way ANOVA with Tukey's multiple comparison test; n=3 independent experiments (each with three technical replicates).

I Immunoblot showing siRNA knockdown of the SRSF1 splicing factor in CHP-134 cells treated with T1-44 (200nM) or DMSO for 72h. Symmetric dimethylation (SDMe) served as a marker for PRMT5 activity and ß-Actin was used as a loading control for this experiment.

J Changes in the differential splicing events of apoptotic genes *DIABLO*, *BCL2L11*, *ACIN1*, and *CFLAR* in CHP-134 cells treated with siSRSF1 or siControl and T1-44 or DMSO for 72 h. Results display the mean inclusion/exclusion ratios for each of the skipped exons ±SD; significance was calculated with a one-way ANOVA with Tukey's multiple comparison test; n=3 independent experiments (each with three technical replicates).

K Immunoblot showing siRNA knockdown of the SRSF4 splicing factor in CHP-134 cells treated with T1-44 (200nM) or DMSO for 72h. Symmetric dimethylation (SDMe) served as a marker for PRMT5 activity and ß-Actin was used as a loading control for this experiment.

L Changes in the differential splicing events of apoptotic genes *DIABLO*, *BCL2L11*, *ACIN1*, and *CFLAR* in CHP-134 cells treated with siSRSF4 or siControl and T1-44 or DMSO for 72 h. Results display the mean inclusion/exclusion ratios for each of the skipped exons ±SD; significance was calculated with a one-way ANOVA with Tukey's multiple comparison test; n=3 independent experiments (each with three technical replicates).

Supplementary Figure 7.

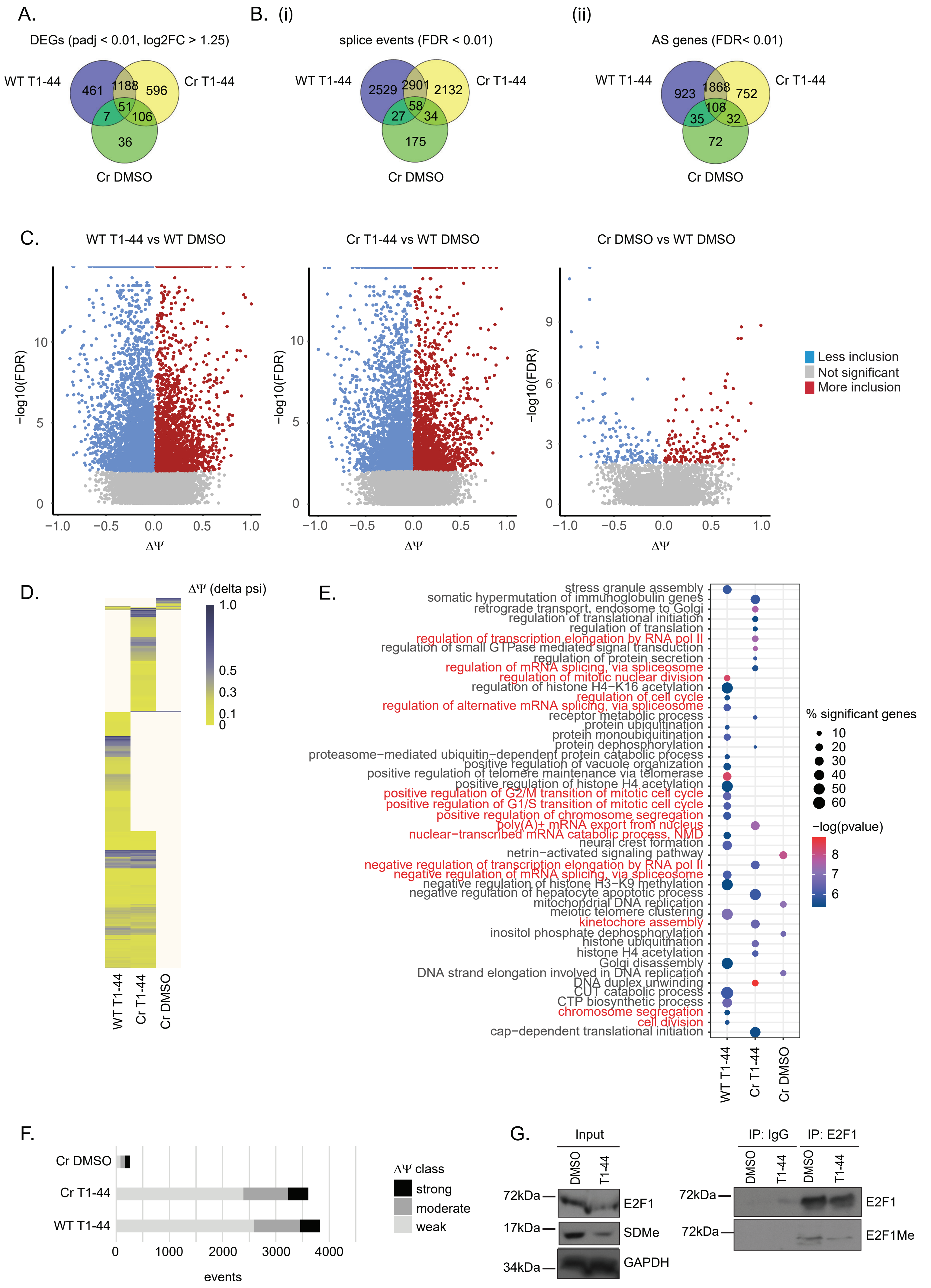

SI Figure 7: Analysis of differentially spliced genes in E2F1 CRISPR CHP-134 cells treated with PRMT5 inhibitor T1-44

A Venn diagram displaying the overlap of differentially expressed genes in each treatment condition, as compared to the wild-type (WT) E2F1 CHP-134 cells treated with DMSO (log_2(FC) > 1.25, padj < 0.01).

B Venn diagrams showing overlap between (i) differential splicing events or (ii) differentially spliced genes identified in each of the treatments, with respect to wild-type (WT) E2F1 CHP-134 cells treated with DMSO.

C Volcano plots displaying values of delta psi ($\Delta\Psi$; percent spliced in) and –log10 FDR for splice events identified in wild-type (WT) E2F1 and E2F1 CRISPR (Cr) CHP-134 cells treated with T1-44 or DMSO, with respect to the WT E2F1 cell line treated with DMSO. Red colour represents increased $\Delta\Psi$ values, whilst blue colour represents decreased $\Delta\Psi$ values. Grey colour represents splice events that fell below the statistical cut-off applied (FDR < 0.01).

D A heat map displaying values of delta psi ($\Delta\Psi$; percent spliced in) in wild-type (WT) E2F1 and E2F1 CRISPR (Cr) CHP-134 cells treated with T1-44 or DMSO, corresponding to statistically significant differential splicing event changes (FDR < 0.01) with respect to the WT E2F1 cell line treated with DMSO. Yellow colour represents the lowest difference, and blue colour represents the highest. Ivory blocks correspond to no significant changes in splicing patterns (FDR > 0.01).

E The topGO package was used to display gene ontology biological process (GO:BP) terms enriched for genes that are alternatively spliced in each treatment condition, as compared to wild-type (WT) E2F1 cells treated with DMSO (FDR < 0.01). Terms related to gene expression, RNA processing, cell cycle transition and mitosis are highlighted in red.

F Differential changes in splicing between the indicated treatments as compared to wild-type (WT) E2F1 cells treated with DMSO. The bar chart displays the total number of splicing events that score as strong ($\Delta\Psi$ > 0.5), moderate ($\Delta\Psi$ between 0.3-0.5), or weak ($\Delta\Psi$ between 0.1-0.3) splice events. Events with a $\Delta\Psi$ < 0.1 were excluded from the analysis.

G A repeat experiment for the E2F1 immunoprecipitation performed in CHP-134 cells displayed in Figure 7A.

Supplementary Figure 8.

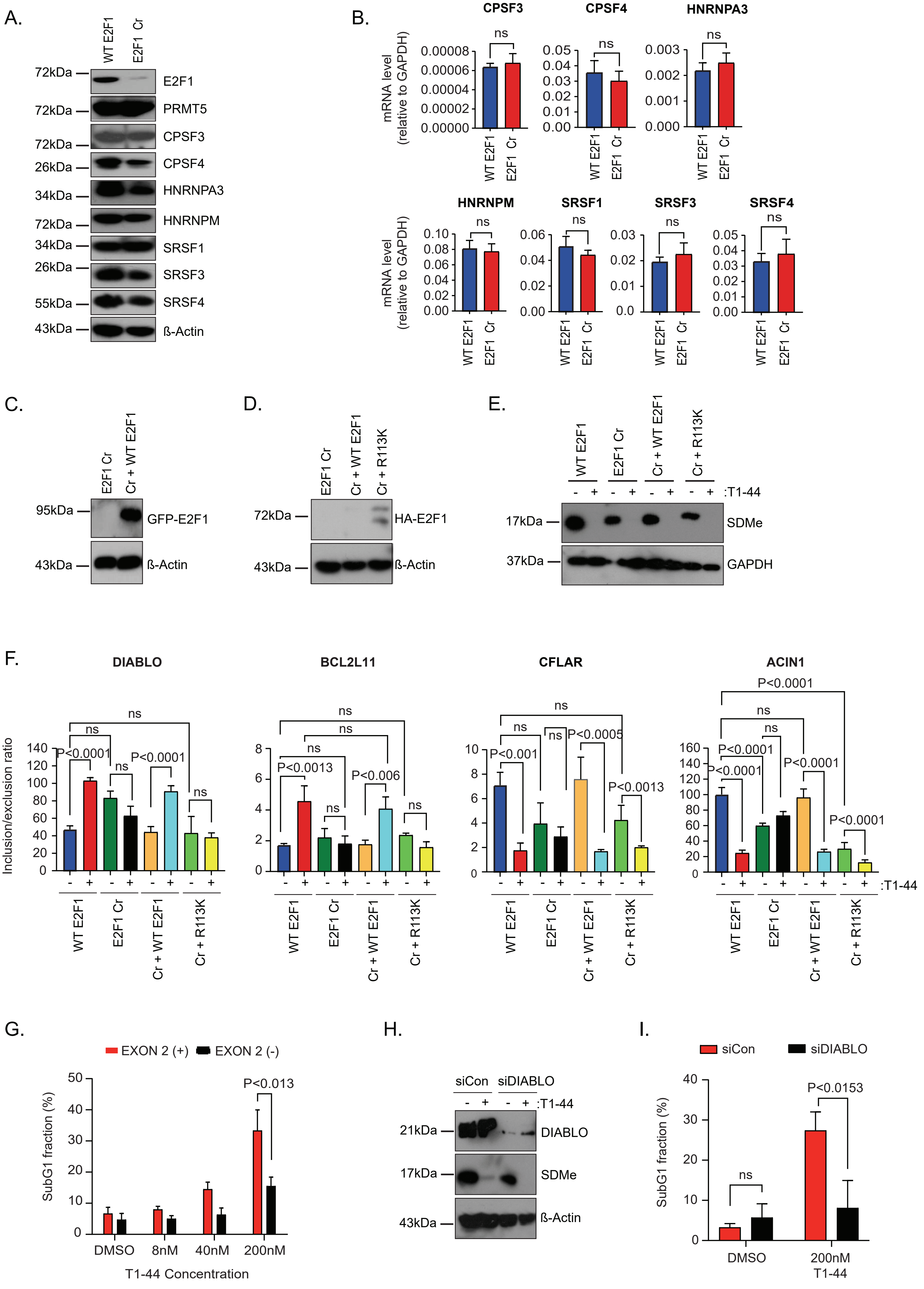

SI Figure 8: Analysis of differential splicing events in E2F1 CRISPR CHP-134 cell lines overexpressing wild-type or methylation-defective E2F1

A Immunoblot to display protein levels of the seven splicing factors (CPSF3-4, SRSF1, 3, 4, HNRNPA3 and HNRNPM) in the wild-type (WT) E2F1 and the E2F1 CRISPR (Cr) CHP-134 cell lines. β-actin served as a loading control for these experiments.

B mRNA expression levels of *CPSF3-4*, *SRSF1*, *3*, *4*, *HNRNPA3* and *HNRNPM* in wild-type (WT) E2F1 and the E2F1 VRISPR (Cr) CHP-134 cell lines. Results represent the mean expression values ±SD; significance was calculated with a one-way ANOVA with Tukey's multiple comparison test; n=3 independent experiments (each with three technical replicates).

C Immunoblot displaying green fluorescent protein (GFP) expression levels in E2F1 CRISPR (Cr2) CHP-134 cells expressing GFP-tagged full-length wild-type E2F1. β-Actin levels served as the loading control.

D Immunoblot displaying haemagglutinin (HA) expression levels in E2F1 CRISPR (Cr2) CHP-134 cells expressing the HA-tagged E2F1 R113K derivative. β-Actin levels served as the loading control.

E Immunoblot displaying symmetric dimethylation (SDMe) levels in wild-type (WT) E2F1 or E2F1 CRISPR (Cr2) cells expressing the HA-tagged E2F1 R113K derivative or GFP-tagged full-length wild-type E2F1, treated with T1-44 for 72 h. GAPDH served as a loading control.

F Changes in the differential splicing events of the apoptotic genes *DIABLO*, *BCL2L11*, *ACIN1*, and *CFLAR* upon the expression of ectopic wild-type E2F1 or E2F1 R113K in the E2F1 CRISPR (Cr) cell lines (Cr + WT E2F1 and Cr + R113K respectively), as indicated, and treated with T1-44 for 72 hrs. For comparison, the analysis is also shown for untransfected WT and E2F1 Cr cells. Results represent the mean inclusion/exclusion ratios for the skipped exon events ±SD; significance was calculated with a one-way ANOVA with Tukey's multiple comparison test; n=3 independent experiments (each with three technical replicates).

G Cell viability upon expressing DIABLO EXON 2+ or EXON 2- in CHP-134 cells and treatment with increasing concentrations of T1-44 (8nM-200nM) for 144 h. The subG1 fraction of cells was measured by propidium iodide staining. Results represent the mean percentage of subG1 cells ±SD; significance was calculated with an unpaired t-test; n=3 independent experiments (each with three technical replicates).

H Immunoblot displaying DIABLO and symmetric dimethylation (SDMe) levels in CHP-134 cells treated with a control SiRNA or siDIABLO following 72 h treatment with T1-44. β-actin served as a loading control for this experiment.

I Cell viability of CHP-134 cells treated with a control siRNA or siRNA against DIABLO, followed by 72 h treatment with T1-44 (200nM). The subG1 fraction of the cells was measured by propidium iodide staining. Results represent the mean percentage of subG1 cells ±SD; significance was calculated with an unpaired t-test; n=3 independent experiments (each with three technical replicates).

Supplementary Figure 9.

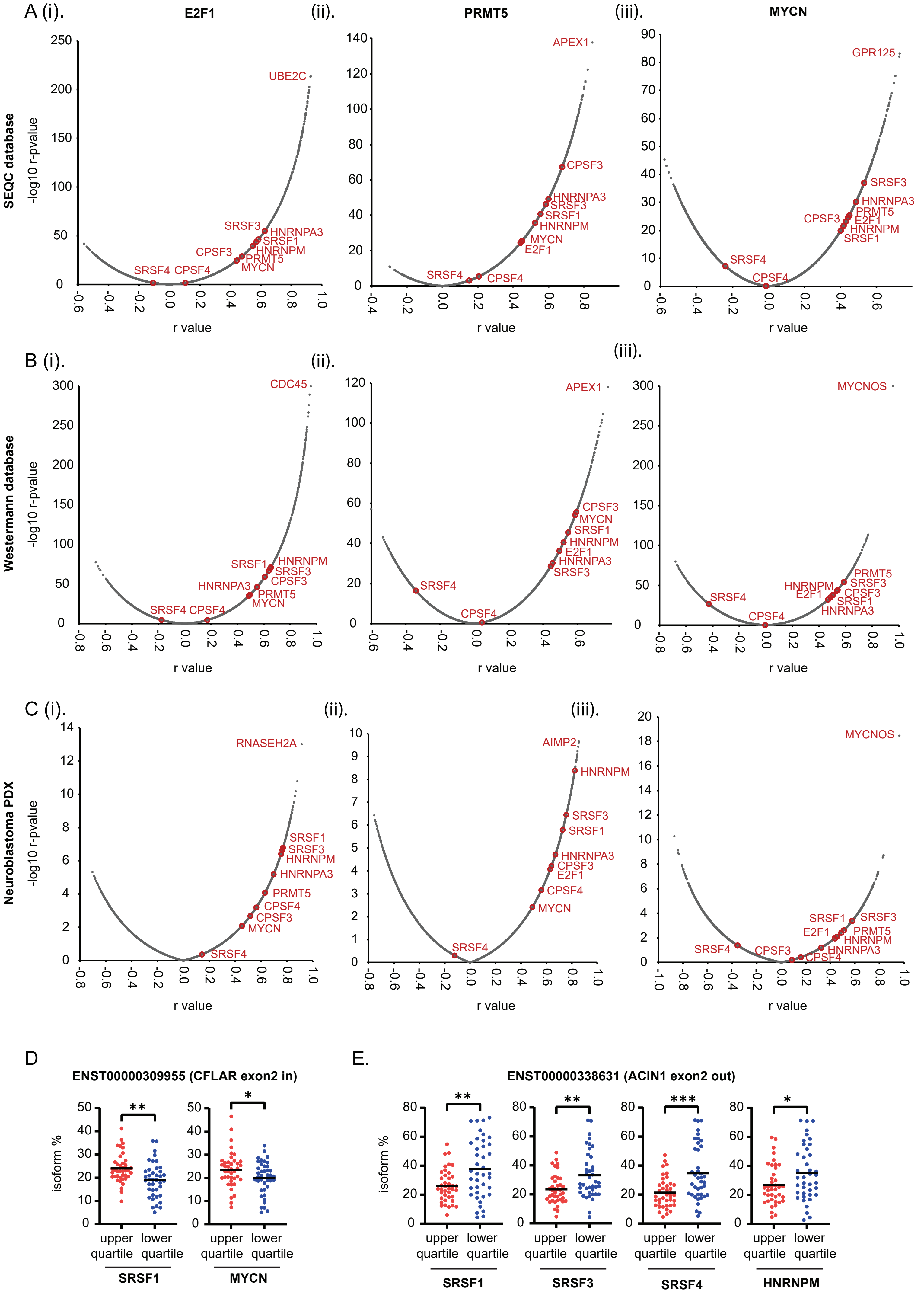

SI Figure 9: Correlation in expression between *MYCN*, *E2F1*, *PRMT5* and splicing factors in human neuroblastoma samples.

A and B Correlation between expression of (i) *E2F1*, (ii) *PRMT5* and (iii) *MYCN* and the indicated seven splicing factors in neuroblastoma patient samples contained within the SEQC (n=498) and Westermann (n=579) Pan-Cancer study. Statistical analysis was performed using ranked Pearson's correlation with an FDR used for correction for multiple testing.

C Correlation between expression of *E2F1*, *PRMT5* and *MYCN* and the indicated seven splicing factors in neuroblastoma PDX models contained within the PPTC dataset (n=33). Statistical analysis was performed using ranked Pearson's correlation with an FDR used for correction for multiple testing.

D Correlation between expression of the splicing factor *SRSF1* and *MYCN* levels in neuroblastoma patient samples within the TARGET (n=161) pan-cancer study, with the expression of an exon 2 containing transcript derived from the *CFLAR* gene, displayed (ENST00000309955). Statistical analysis was performed using a student's t-test with Welch's correction.

E Correlation between the expression of the splicing factors *SRSF1, 3, 4* and *HNRNPM* and the expression of an exon 2 excluded transcript derived from the *ACIN1* gene is displayed (ENST00000338631). Statistical analysis was performed using a student's t-test with Welch's correction.

Table S1: Details of all primer sequences used in this study.

Table S2: A list of neuroblastoma cell lines used in this study, and a summary of their corresponding *MYCN*, *PRMT5* and *E2F1* mRNA expression levels and IC_{50} values in response to T1-44 treatment.

Table S3: DEG analysis on CHP-134 and GI-ME-N cells treated with T1-44 (compared to DMSO).

Table S4: rMATS differential splicing analysis on CHP-134 and GI-ME-N cells treated with T1-44 (compared to DMSO).

Table S5: DEG analysis of CHP-134 WT E2F1 and E2F1 Cr cells treated with T1-44 (compared to WT E2F1 cells treated with DMSO).

Table S6: rMATS differential splicing analysis of CHP-134 WT E2F1 and E2F1 Cr cells treated with T1-44 (compared to WT E2F1 cells treated with DMSO).

Table S7: TARGET neuroblastoma sample expression data.
